# Bacteria forming drag-increasing streamers on a drop implicates complementary fates of rising deep-sea oil droplets

**DOI:** 10.1038/s41598-020-61214-9

**Published:** 2020-03-09

**Authors:** Andrew R. White, Maryam Jalali, Michel C. Boufadel, Jian Sheng

**Affiliations:** 1 Department of Engineering, Texas A&M University–Corpus Christi, Corpus Christi, TX 78412 USA; 2 Center for Natural Resources, Department of Civil and Environmental Engineering, New Jersey Institute of Technology, Newark, NJ 07102 USA

**Keywords:** Marine biology, Mechanical engineering

## Abstract

Competing time scales involved in rapid rising micro-droplets in comparison to substantially slower biodegradation processes at oil-water interfaces highlights a perplexing question: how do biotic processes occur and alter the fates of oil micro-droplets (<500 *μm*) in the 400 m thick *Deepwater Horizon* deep-sea plume? For instance, a 200 *μm* droplet traverses the plume in ~48 h, while known biodegradation processes require weeks to complete. Using a microfluidic platform allowing microcosm observations of a droplet passing through a bacterial suspension at ecologically relevant length and time scales, we discover that within minutes bacteria attach onto an oil droplet and extrude polymeric streamers that rapidly bundle into an elongated aggregate, drastically increasing drag that consequently slows droplet rising velocity. Results provide a key mechanism bridging competing scales and establish a potential pathway to biodegradation and sedimentations as well as substantially alter physical transport of droplets during a deep-sea oil spill with dispersant.

## Introduction

After the *Deepwater Horizon* (DWH) oil rig explosion, millions of barrels of oil were released from the seafloor wellhead at a depth of 1500 m^1–3^. A significant amount of research has been devoted to determining the fate of the spilled oil and their mechanisms. It was estimated that close to half of the oil rose to the surface as millimeter (mm-) scale droplets^1,4^ within hours, while much of the remaining hydrocarbons formed a deep-sea plume between depths of 900 and 1300 m^3,5–8^. Among these hydrocarbons trapped in the plume, oil droplets containing insoluble hydrocarbons such as *n-*alkanes in the form of *μ*m-scale droplets (<500 *μm*) were directly observed for the first time^9^. This unprecedented generation of micro-scale oil droplets was facilitated by the wellhead injection of 2.9 million liters of dispersant^10^ that lead to substantial reduction of interfacial tensions at the oil water interfaces, promoting breakup of larger oil droplets into a pool of micro-scale droplets as demonstrated experimentally by Gopalan & Katz^11^. Recent models likewise suggest such prolific production of micro-scale droplets is not possible without dispersant^12^, and the presence of the dispersant’s active ingredient in the plume^13^ further supports dispersants’ role in forming a microdroplet-laden plume. While these droplets are estimated to have accounted for 13–43% of the total plume mass^4^, their fate is a matter of debate in the scientific community^14^.

Since the deep-sea plume coincided spatially and temporally with a microbial bloom^5,15–23^, conventional wisdom suggests biodegradation as the primary fate of these micro-scale plume droplets. Laboratory simulations of the deep-sea plume using chemically dispersed 10 *μm* droplets show however half-lives of *n-*C_6_ to *n-*C_25_ alkanes are 6–8 d with an initial lag of 5–10 d^24^. Considering a 100 *μm* droplet in the plume (assuming fresh oil in 4 °C seawater) rises through a 100 m depth (a typical maximum thickness of microbial bloom patches^5,15–22^ within the plume) in ~3 d, it is too short for substantial biodegradation processes on *n*-alkanes to occur, i.e. to successfully degrade an oil droplet, microbes must “reside” in close vicinity of the droplet for a longer time. Without a mechanism capable of slowing down a rising droplet through this microbial bloom, biodegradation would be severely limited.

Additionally, field sediment measurements have demonstrated an oil sedimentation event accounting for 2–14% of the total released oil that is temporally synchronized with events of the spill and spatially co-located underneath the plume^7,25–30^. Compounded with the distribution of hopane in sediments, Valentine *et al*.^7^ have concluded that the deep-sea plume is the major source of hydrocarbon sedimentation and consequently identify sedimentation as one of the crucial fates for droplets in the plume. One potential mechanism responsible for this sedimentation event is Marine Oil Snow Sedimentation and Flocculent Accumulation (MOSSFA)^31^, wherein sticky marine snow flocs facilitated by planktonic secretions (Extracellular Polymeric Substances or EPS)^32–34^ laced with oil droplets (Marine Oil Snow or MOS^35–38^) sediment to the seafloor. But again, the short residence time of an oil droplet rising through the microbial bloom would presumably prevent initiation of MOS in the plume. A mechanism capable of bridging the short residence time of a rising droplet and the long interaction times with the microbial bloom necessary to form MOS has not been identified due in part to the difficulty in conducting experiments in both relevant length (e.g. *μ*m) and time scales (e.g. days or weeks).

To circumvent the difficulties in observing a rising oil micro-droplet in a bacterial suspension, we have developed a closed-loop microfluidic microcosm (*Ecology-on-a-Chip* or *eChip*) wherein a *stationary* oil droplet in a microfluidic channel is subjected to a continuous flow containing a bacterial suspension^39^. The *eChip* emulates the biological, chemical and hydrodynamic microcosm environment around a rising droplet at ecologically relevant time scales (days) while simultaneously observing bacterium-droplet interactions at individual cellular scales (1 ms and 1 *μ*m). Bacteria are cultivated *in situ* allowing uninterrupted long-term experimentation. Simultaneous high speed imaging and time lapse microscopy provide direct observations of bacteria-droplet interactions at the oil water interface.

Bacteria are known to form aggregates (e.g. biofilms) and filamentous structures (e.g. streamers) on solid-liquid interfaces under flow conditions. Originally thought to only occur in turbulent flow conditions^40^, it has been demonstrated recently using microfluidics that streamers can also form in laminar flows^41^. It has been shown that streamers in laminar flows are formed through two primary pathways: either by precursor EPS threads created by an established bacterial film, which crosses flow streamlines and recruits passing suspended bacteria^41–45^, or by pre-formed bacterial flocs from upstream that attaches to the solid surface and are rapidly sheared to form streamers^46^. More recently, we have shown for the first time that bacterial aggregates and streamers can be formed directly on a sheared oil-water interface^39^. Using the same *eChip* platform, we demonstrate here that streamers form on a rising oil drop by first developing precursor threads which cross streamlines and trap additional bacteria as they pass by. The attached colony undergoes a “life cycle” analogous to that of biofilms observed at solid-liquid interfaces. Similar to their counterparts at solid-liquid interfaces^41–44,46^, these streamers formed over a droplet substantially increase its drag (e.g. at least 80% increase by only two streamers). Experiments also reveal that within an hour, isolated streamers bundle into a robust tail that can extend more than 10 drop diameters downstream and cause a further increase in drag. Such a rapid increase in drag would drastically reduce the rising velocity of a droplet and consequently enhance residence times of bacteria near the droplet, leading to oily micro-flocs by further accumulation of bacteria and EPS. Anecdotally, we have also observed attachment of streamers to nearby droplets in the *eChip* environment as a precursor to MOS. Thus, the formation of streamers directly on a rising oil droplet, which increases drag and reduces its rising velocity, establishes a plausible mechanism for MOS formation directly in the deep-sea plume, possibly leading to both sedimentation as well as improving bacterial residence times required for biodegradation of oil droplets that would have otherwise risen to the surface.

## Results and Discussion

### Formation of MOS around a single rising droplet

**Figure 1:**
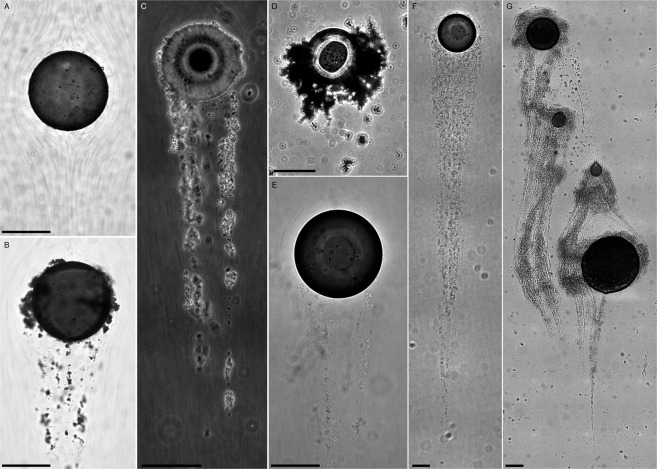
Gallery of a single oil droplet in flows with various dense suspensions containing (**A**) sterile 1 *μ*m latex beads (${10}^{8}\,{\rm{bds}}\cdot {{\rm{ml}}}^{-1}$) in DI water ($R{e}_{{D}_{d}}=0.4$); (**B**) sterile 1 *μ*m latex beads (${10}^{8}\,{\rm{bds}}\cdot {{\rm{ml}}}^{-1}$) with minor bacterial contamination ($O{D}_{600} < 0.01$) in DI water ($R{e}_{{D}_{d}}=0.4$); (**C**) sterile 1 *μ*m latex beads (${10}^{8}\,{\rm{bds}}\cdot {{\rm{ml}}}^{-1}$) spiked with *Sagitulla stellata* ($O{D}_{600} < 0.01$) in sterilized Artificial Seawater (ASW, 25 ppt) with purified Extracellular Polymeric Substances (EPS, $10\,{\rm{mg}}\cdot {{\rm{l}}}^{-1}$) from *Sagitulla stellata* ($R{e}_{{D}_{d}}=0.2$); (**D**) sterile 1 *μ*m latex beads (${10}^{8}\,{\rm{bds}}\cdot {{\rm{ml}}}^{-1}$) in ASW (25 ppt) with purified EPS ($1\,{\rm{mg}}\cdot {{\rm{l}}}^{-1}$) from natural Gulf of Mexico microbial assemblage ($R{e}_{{D}_{d}}=0.2$); (**E-G**) *in situ* cultured *Pseudomonas sp* (strain P62, $0.35 < O{D}_{600} < 0.56$) in nutrient broth. Time lapsed micrographs of an oil droplet passing through *Pseudomonas* suspension recorded at Δ*t* = 16 min (**E**) and 50 min (**F**) immediately after the droplet encounters bacterial suspension, when several streamers are initiated (**E**) and a long streamer bundle is formed (**F**) respectively ($R{e}_{{D}_{d}}=0.4$). (**G**) Multiple pinned droplets are interconnected by a web of EPS aggregates and streamers to form a larger oily aggregate after 120 h exposure to *Pseudomonas* (P62) suspension ($R{e}_{{D}_{d}}=1.6$). Flow is downward in all panels. All images are taken using differential interference contrast (DIC) except for (**C**) with phase contrast. Scale: 100 *μ*m.

Using the *eChip* platform, we conduct long-term microcosm experiments lasting ~3 d to demonstrate first that EPS aggregates (*i.e*. EPS, cells, and particles) can form at a sheared oil-water interface, such as around a rising oil droplet (Fig. 1). Before each microcosm experiment (conditions summarized in Table 1), a single ~180 *μ*m crude oil droplet (${D}_{d}=183\pm 46\,\mu {\rm{m}}$) is generated and pinned in a microchannel (*μ*channel) with a dimensions of $60\,{\rm{mm}}\,(L)\times 11\,{\rm{mm}}\,(W)\times 100\,\mu {\rm{m}}\,(H)$. While the droplet is pinned to the top and bottom channel walls, the oil-water interface of the droplet is initially mobile (Video S1). The imaging plane (depth of field ~5 *μ*m) is situated at the mid-plane of the channel far away from both walls to minimize near wall effects. The pinned droplet is subjected to a flow driven by a peristaltic pump at speeds close to its Stokes rising velocity, ${U}_{d}=({\rho }_{d}-{\rho }_{f})g{D}_{d}^{2}/18{\mu }_{f}$, where *ρ*_*d*_ and *ρ*_*f*_ are the density of the droplet and the surrounding fluid, and *μ*_*f*_ is the surrounding fluid dynamic viscosity. For clarity, hereinafter subscript “*d*” refers to the droplet and “*f*” to the surrounding fluid. Note that all experiments are performed at 20 °C at which temperature the following analysis is performed. We use hydrocarbon degrader *Pseudomonas sp*. (strain P62, ATCC 27259^47^) culture as our model system in kernel experiments as well as various suspensions of sterilized 1 *μ*m latex beads (Duke Scientific, ${10}^{8}\,{{\rm{ml}}}^{-1}$, Fig. 1A), latex beads with bacterial cells at low concentration (optical density at 600 nm wavelength $O{D}_{600} < 0.01$, Fig. 1B), and sterile (i.e. no living bacteria present) extracted EPS ($1\,{\rm{to}}\,10\,{\rm{mg}}\,{{\rm{l}}}^{-1}$, Fig. 1C,D) in auxiliary experiments to highlight the study’s ecological implications. In kernel experiments (Fig. 1E–G), a *Pseudomonas* suspension is inoculated and cultured *in situ* within the *eChip*. When the culture reaches its mid-log growth ($O{D}_{600}\approx 0.4$), the suspension is allowed to flow through the μchannel containing the pinned oil droplet, emulating the scenario that a rising droplet encounters an ocean layer rich with microbes.

**Table 1:** Experimental conditions for microcosm experiments as discussed in the maintext with their corresponding figures.

*Exp*.		Oil droplet characteristics			Flow	Particulate suspension characteristics			
*ID*	*Fig*.	Oily phase medium	Size *D*_*d*_(*μm*)	Stokes speed *u*_*d*_(*mm* / *s*)	Flow *U*_*f*_ / *u*_*d*_	Suspension medium (Duke Scientific)	_1 *μm*_ Beads (Bds/ml)	Microbes (*OD*_600_)	
E1	1 A	Macondo surrogate crude (MC)	170	1.5	1.67	DI water (<1ppm SDS)	10^8^	No bacteria	
E2	1B		170	1.5	1.46			Contamination (*OD* < *0.01*)	
E3	1 C		122	1.15	1.6	ASW (25*ppt*) + *Sagittula* EPS (1 *mg*·*l*^−1^)		*Sagittula stellate* (*OD* < *0.01*)	
E4	1D		154	1.23	1.0	ASW (25ppt) +GOM consortia EPS (1 *mg*·*l*^−1^)		No bacteria	
E5	1E-F, 2		175	2.74	1.3	Difco nutrient broth medium (8 *g*·*l*^−1^) with no additional salt	N.A.	*Pseudo-monas sp*. (P62)	OD
									0.35
E6	1 G	MC + 9500 A (0.1% v/v)	250	3.24	2.8				0.56
E7	3,4,5	MC	240	2.99	0.74				0.41

ASW: sterilized Artificial Seawater. Stokes rising velocity is calculated using ${u}_{d}=(SG-1)g{D}_{d}^{2}/(18{\nu }_{f})$, where *SG* is the specific gravity for oil to surrounding fluids, ${\rho }_{oil}/{\rho }_{f}$, and *v*_*f*_ is the kinematic viscosity. All estimations in this table are performed at 20 °C, and *SG* is assumed to be 0.9 for a slightly weather oil.

Kernel experiments (Fig. 1E–G) reveal that as a droplet encounters a dense *Pseudomonas* suspension ($\sim {10}^{8}\,{\rm{cells}}\,{{\rm{ml}}}^{-1}$), within tens of minutes (e.g. 16 min in Fig. 1E) several thin EPS filaments containing bacteria are rapidly formed behind the droplet. Note that these apparently thin filaments (≪1 *μ*m in diameter) are transparent and only observable via attached bacterial cells. Each filament with one end anchored directly to the rear of the oil droplet extends at least 2 *D*_*d*_ downstream. These observations of filaments at a *liquid-liquid* interface draw parallels to the similar phenomenon occurring at a solid-liquid surface during the formation of a biofilm, *e.g*. “streamers”^42–44,46^. To emphasize their intrinsic similarity, we hereon refer to these filaments as “streamers”, i.e. elongated thin EPS threads attached randomly with bacterial cells or particles.

As time progresses, these individual streamers are bundled together to form a prominent tail extending 12*D*_*d*_ downstream (Fig. 1F). Focusing periodically on the top and bottom channel walls confirm that these streamers are formed at the mid-plane of the channel and not initiated from walls. As these streamers are forming and bundling around a dense pool of droplets, these polymeric tails encroach upon and connect to nearby droplets (Video S2) to form large mm-scale oily MOS particles demonstrated anecdotally in Fig. 1G. Here a network of four oil droplets is interconnected with streamer bundles forming a web of bacteria, EPS and oil droplets*, i.e*. a small MOS particle. These robust and rapid formations of streamers and streamer bundles in a matter of hours will have significant impacts on drag as discussed later, and the formation time scales are sufficiently short to substantially affect the rising velocity of oil micro-droplets as they encounter microbial blooms in a deep-sea plume.

We have conducted several auxiliary experiments (E1–E4 in Table 1, Fig. 1A–D) to identify key components in these processes. A control experiment using sterilized seawater (25 ppt) with 1 *μ*m latex beads at ${10}^{8}\,{\rm{bds}}\cdot {{\rm{ml}}}^{-1}$ reveals that, except for sporadic adsorption of single beads, no aggregation is observed (Fig. 1A). However, an experiment using the same 1 *μ*m latex bead concentration but with bacterial contamination ($O{D}_{600} < 0.01$) yields streamers initiated on the droplet surface in less than 60 min which bundled together to form a tail after 11 h shown in Fig. 1B. This demonstrates that significant particle aggregation on the oil droplet requires secretions from bacteria. To further identify what materials produced by bacteria would initiate streamers, we conduct experiments using two sterilized particle suspensions (${10}^{8}\,{\rm{bds}}\cdot {{\rm{ml}}}^{-1}$) containing (i) 10 ${\rm{mg}}\cdot {{\rm{l}}}^{-1}$ “non-attached” EPS purified from *Sagittula stellata* culture^48^ spiked with live *Sagittula* cells (*OD*_600_ ≪ 0.01 or $1000\,{\rm{cells}}\cdot {{\rm{ml}}}^{-1}$) (Fig. 1C) and (ii) 1 ${\rm{mg}}\cdot {{\rm{l}}}^{-1}$ EPS purified from natural assemblage collected near the DWH site (Fig. 1D). In both cases, particles aggregated around the droplet, while streamers are only formed in the suspension containing live *Sagittula* (Fig. 1C). We can conclude that with EPS, a MOS aggregate composed of particles, cells and oil can be readily formed directly on a rising crude oil droplet in less than 1 h. Formation of streamers, however, appears to require the presence of live bacteria independent of species (e.g. garden variety of bacterial contaminations in Fig. 1B and *Sagittula* in Fig. 1C) and even at extremely low concentrations (e.g. <1,000 cells ml^−1^ in Fig. 1B,C, Table 1). Although details are still unknown, a minute number of live bacteria does appear to improve the elasticity of EPS and consequently is more prone to form streamers. These experiments support the assertion that streamer initiation and bundling is a mechanism robust enough to occur in real ocean environments such as during the DWH oil spill. In the following, we will further substantiate these assertions with discussions on the evolution of the streamers and its direct impacts on hydrodynamics.

### “Life cycle” of a streamer bundle behind a rising droplet

**Figure 2:**
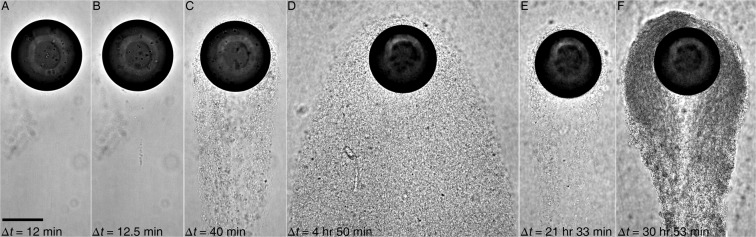
Micrographs of a crude oil drop in a flow containing *Pseudomonas sp*. (P62) at various times immediately after its exposure to bacterial suspension, Δ*t*, of (**A**) 12 min, (**B**) 12.5 min, (**C**) 40 min, (**D**) 4 h 50 min, (**E**) 21 h 33 min, and (**F**) 30 h 53 min. They show stages of EPS streamers around an oil drop as (**A**) smooth drop, (**B**) streamer initiation, (**C**) bundling, (**D**) proliferation and aggregation, (**E**) dispersal, and (**F**) reformation. The flow ($R{e}_{{D}_{d}}=0.4$) is downward in all panels. Scale: 100 *μ*m.

We use *Pseudomonas sp*. as the model system to elucidate the temporal evolution of a streamer bundle formed behind a rising droplet (Fig. 2). Although *Pseudomonas* is a motile hydrocarbon degrader^21^, our prior studies do not recover any specific taxis behaviors to crude oil nor any significant evidence of its motility impacting surface adsorption (*SI* S.1). Hence, we expect the following observations to be generic to any bacteria near an oil drop. Figure 2 shows the time evolution of a streamer bundle (Video S3) at $\Delta t=0.2,\,0.208,\,0.667,\,4.83,\,21.55,\,{\rm{and}}\,30.883\,{\rm{h}}$ immediately after the droplet’s exposure to dense *Pseudomonas* culture ($O{D}_{600}=0.35$). The mean flow speed is ${U}_{f}=3.6\,{\rm{mm}}\,{{\rm{s}}}^{-1}$ into the μchannel where a micro droplet (${D}_{d}=175\,\mu {\rm{m}}$) is pinned.

*Pseudomonas* containing EPS encounter the droplet at the leading edge and are driven by flow shear towards its trailing edge. Cells with EPS are quickly launched into the flow with one end firmly anchored at the oil-water interface, forming a streamer. The EPS streamer connecting cells is further stretched by flow shear and extruded to several drop diameters (*D*_*d*_) downstream (Fig. 1E). The streamer initiation is a rapid process that completes within 30 s demonstrated in Fig. 2A (no streamer) to Fig. 2B (with a streamer 30 s later). Since a single streamer is apparently relatively weak, it detaches easily from the droplet. This initiation and break-off of streamers occurs periodically within the first 30 min, after which streamers bundle to form a robust tail (Fig. 2C at 40 min after initial exposure). This bundle covers the entire drop surface and extends up to >10 *D*_*d*_ downstream (Fig. 1F). Bacteria “trapped” in this polymer matrix are individually identifiable (bright rods with dark edges in Fig. 2C). Within several hours, the bundle evolves into a large oily EPS aggregate with a width of 3 *D*_*d*_ and a length of 20 *D*_*d*_. Note that it takes only 4 h 50 min to reach its maximum size and proliferation of cells (Fig. 2D). These timescales are similar to those reported for streamer formation on solid microchannel walls^41^.

Analogous to the “dispersion” phase of a mature biofilm over a solid surface^49^, we have also observed the dispersal of aggregates in streamer bundles and return to a thin polymer “shroud” covering the entire droplet (Fig. 2E). Note that the dispersal process took 15 h in Fig. 2. Further examination of time-lapsed recording (Video S3) reveals that the process is composed of “erosion dispersal” at the outer edge and “seeding dispersal” (i.e. central hollowing^49^) at the center of the tail. For traditional biofilms, “erosion dispersal” can be either an active bacterial process or passive (i.e. flow shear) process, whereas “seeding dispersal” is always an active process^49^. This observation of active bacterial dispersal following initial colonization of an oil droplet has significant implications on bacterial degradation process in the water column. A short time later, a much more robust tail is re-formed (e.g. 9 h later in Fig. 2F). Note that the matrix in Fig. 2F is denser and cell concentration is higher than those in Fig. 2D. Although Fig. 2 only illustrates a single experiment, the abovementioned processes and their associated time-scales have been confirmed and validated by additional five duplicated experiments. We conclude that not only do polymeric aggregates consisting of cells, particles and EPS form directly on an oil droplet surface, but also the formation process involves very drastic morphological changes and complex interactions by the nearby microbial community under flow shear via cell attachment (Fig. 2A,B), streamer initiation (Fig. 2B), bundling (Fig. 2C), proliferation/growth (Fig. 2D), dispersal (Fig. 2E) and regrowth (Fig. 2F).

### Hydrodynamic impact of streamers on the rising velocity of a droplet

To address the streamers’ hydrodynamic impact, we measure hydrodynamic drag on an oil droplet with attached streamers directly. The experiment was conducted in the *eChip* microcosm at room temperature (20 °C) using model bacterium *Pseudomonas* (P62). After reaching mid-log growth ($O{D}_{600}=0.41$), the dense bacterial suspension is allowed to flow into the observation μchannel where a 240 *μ*m drop is pinned at ${U}_{f}=2.2\,{\rm{mm}}\,{{\rm{s}}}^{-1}$ (or ${U}_{f}=0.74{U}_{d}$). Mean flow fields are measured at the mid-plane of the channel by a high speed camera at an interval of 10 min for several days.

**Figure 3:**
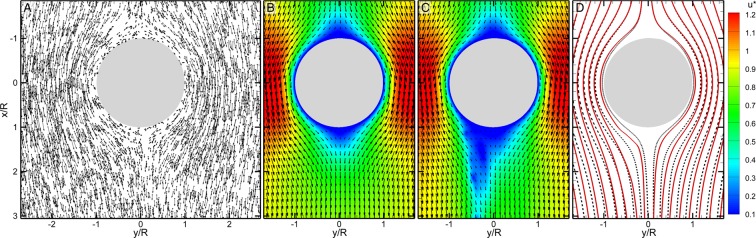
Flow measurements around an oil droplet with and without trailing streamers. (**A**) Sample instantaneous tracer particle velocity (only 0.5% of total $\sim 3\times {10}^{6}$ vectors are shown). Dot: location of the cell, Arrow: velocity. Mean flow velocity fields around (**B**) a smooth droplet (Δ*t* = 20 min min) and (**C**) a droplet with two streamers (Δ*t* = 30 min) superimposed onto their velocity magnitudes (colored contour). Each mean field is averaged over 999 instantaneous realizations. (**D**) Superimposed streamlines from (**B**) – black dotted lines and (**C**) – red solid lines showing the hydrodynamic impact of single or several streamer filaments on flow around a drop. The flow ($R{e}_{{D}_{d}}=0.4$) is in the positive *x*-direction.

A sample flow field around a smooth drop composed of 0.5% of total velocity measurement realizations near the start of the experiment ($\Delta t=20$ min after initial exposure to bacteria) before streamers have formed is shown in Fig. 3A. This unstructured velocity vector (totaling ~$3\times {10}^{6}$ vectors per measurement) are ensemble averaged and interpolated onto a structured grid with a vector spacing of 2.7 *μ*m using a Taylor expansion scheme^50^ to obtain highly resolved mean flow fields (Fig. 3B–D). Mean velocity fields are normalized with the mean flow speed, *U*_*f*_, of the channel. Mean flow fields around a drop with two trailing streamers (Fig. 3C) and without streamers (Fig. 3B) are shown as vector maps (displaying only every 7 in *x*- and every 5 vectors in *y*-axis) superimposed on their corresponding velocity magnitude fields (colored contours). Note that in the high-speed sequence (Video S4) that produces mean flow (Fig. 3C) around a droplet with two streamers, one can only identify two invisible streamers by the attached bacterial clusters that are oscillating in the flow. This also distinguishes the formation pathway as that of precursor EPS threads^41^ rather than by pre-formed flocs from upstream^46^.

Flow around a smooth drop (Fig. 3B) at Δ*t* = 20 min after exposure to bacteria demonstrates classic Stokes flow (i.e. symmetric front to back) around a circular profile. In contrast, the flow captured at Δ*t* = 30 min (Fig. 3C) and containing two trailing streamers shows a clearly developed “wake”, i.e. pronounced “blue” region in Fig. 3C. Note that these localized areas of low flow correlate directly to the presence of EPS streamers. To highlight the hydrodynamic impact of these streamers, we superimpose these two fields on top of each other using streamline pairs (Fig. 3D) where the dashed lines represent the flow in Fig. 3B (no streamer) and the solid red lines represent the flow in Fig. 3C (two streamers). Each pair of streamlines are initiated at the same upstream location. As evident in Fig. 3D, streamlines around a drop with streamers deviate substantially from those from a smooth drop. Although observed in the upstream region (y/R < 0), deviation is more pronounced behind the drop. This apparent widening of the spacing between two adjacent streamlines behind a drop with streamers causes the development of an apparent “wake”.

We emphasize that at the current flow regime ($R{e}_{{D}_{d}}=0.4$, where $R{e}_{{D}_{d}}={\rho }_{f}{U}_{f}{D}_{d}/{\mu }_{f}$ is the Reynolds number), the “wake” is unexpected and uncharacteristic. The substantial loss of fluid momentum behind a drop with streamers compared to a smooth drop compounded by the presence of a “wake” demonstrate that streamers, even only a couple, will greatly impact the hydrodynamic drag on the drop and subsequently reduce its rising velocity drastically. In the following, we quantify the hydrodynamic impact of the streamers by directly estimating drag force on a drop. To accentuate the impact of isolated streamers on drag and subsequently the rising velocity of an oil drop with them, we focus our analysis on the first 100 minutes of our experiment when isolated streamers are formed, detached and reformed.

**Figure 4:**
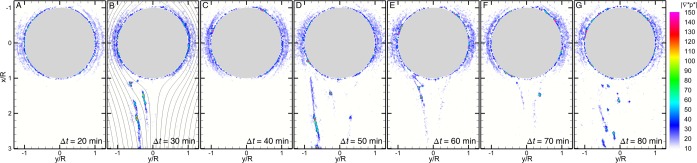
Distributions of normalized pressure gradient magnitude, $|{\nabla }^{\ast }{{\boldsymbol{p}}}^{\ast }|$, around an oil drop in the first 80 min immediately after exposure to *Pseudomonas* (P62) at the time, Δ*t*, of (**A**) 20, (**B**) 30, (**C**) 40, (**D**) 50, (**E**) 60, (**F**) 70, and (**G**) 80 min. The filamentary regions with elevated pressure gradient magnitudes highlight instantaneous locations and shapes of streamers as they are initiated over and detached from the drop sporadically. Streamlines are superimposed in (**B**) to illustrate the crossing of streamers by streamlines. The flow ($R{e}_{{D}_{d}}=0.4$) is in the positive *x*-direction.

With high resolution mean velocity fields resolved at every 10 minutes, we directly estimate the drag on the drop with and without streamers by performing a control volume analysis (*SI* S.2) of steady *x*-axis momentum balance:

1 $$R{e}_{{D}_{d}}({\overrightarrow{u}}^{\ast }\cdot {\overrightarrow{\nabla }}^{\ast }){\overrightarrow{u}}^{\ast }+{\overrightarrow{\nabla }}^{\ast }{p}^{\ast }-{\overrightarrow{\nabla }}^{\ast }\cdot {\overline{\overline{\tau }}}^{\ast }=0,$$

where the superscript “*” denotes the normalized quantities or operators, “∇” is the gradient operator, and ${\overline{\overline{\tau }}}^{\ast }=[{\overrightarrow{\nabla }}^{\ast }{\overrightarrow{u}}^{\ast }+{({\overrightarrow{\nabla }}^{\ast }{\overrightarrow{u}}^{\ast })}^{T}]$ is the normalized viscous stresses. Lengths are scaled by *D*_*d*_, velocities by *U*_*f*_, and stresses by ${\mu }_{f}{U}_{f}/{D}_{d}$. Briefly, the first term is the momentum deficit, the second represents the pressure gradient, and the third viscous stresses including shear (causing skin friction and streamer extension) and normal stress (causing pressure drag and bending of the streamer). Since both momentum (1^st^) and viscous stress (3^rd^) terms are sufficiently evaluated using velocity measurements, the often elusive pressure gradient (2^nd^ term in Eq. 1) is estimated directly. Distributions of normalized pressure, ${p}^{\ast }=p/(2\mu {U}_{f}{D}_{d}^{-1})$, gradient magnitude are shown in Fig. 4 at a time interval of 10 min for the first 80 min of the experiment. As evident in Fig. 4, although a single streamer is thin, transparent and unidentifiable in microscopic images (Video S4), a single filament leaves a clear footprint in the pressure gradient fields, i.e. regions with elevated values collocated with the streamer (Fig. S2).

Figure 4A shows the distribution of the pressure gradient magnitude around a smooth drop with its corresponding flow (Fig. 3B). As expected, the pressure gradient is concentrated around the drop. In 10 min, two streamers with large pressure gradient are extruded from the left side of the drop and elongated in *x* direction (Fig. 4B and corresponding flow in Fig. 3C). Note in Fig. 4B that these streamers clearly do not follow the streamlines (lines in Fig. 4B) behind a drop, but cross them due to the intrinsic elasticity of streamers theoretically predicted by Autrusson *et al*.^51^. At this early stage, streamer filaments are thin and have yet to form bundles. As shown in Fig. 4C, the two streamers observed in Fig. 4B 10 min earlier has since been detached and the flow is recovered. This cyclic process of streamer formation and detachment persists throughout the early stage of the drop encountering the bacteria suspension, i.e. a streamer in the previous frame rarely survives to the next (Fig. 4). As time progresses, streamers increase in number and survive longer by bundling together (Fig. 4G).

**Figure 5:**
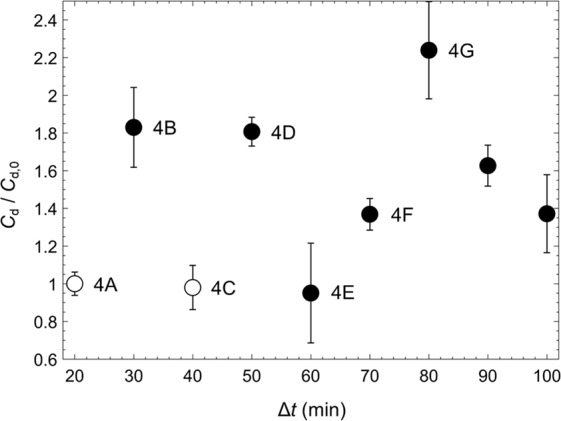
Time evolution of mean drag coefficient, *C*_*d*_, normalized by that of a smooth droplet, *C*_*d*,0_ for Δ*t* = 20 to 100 min. Annotations indicate corresponding $|{\nabla }^{\ast }{p}^{\ast }|$ contours from Fig. 4. Hollow circle: no streamers; Filled circle: with streamer(s); Error bars: one standard deviation from the mean is calculated from the 25 control volume variations per time instance. $R{e}_{{D}_{d}}=0.4$.

To estimate hydrodynamic drag on a drop with streamers, we have performed an analysis by balancing the momentum deficit, pressure forces and viscous stresses on a control volume enclosing both drop and trailing streamers (details in *SI* S.2). Briefly, enclosing the droplet in a control volume with a control surface *S*, we determine the drag force by balancing total forces and momentum flux as the following:

2 $${F}_{d}^{\ast }=-{\int }_{S}[R{e}_{D}\,(\overrightarrow{n}\cdot {\overrightarrow{u}}^{\ast }){u}_{x}^{\ast }+{n}_{x}{p}^{\ast }-\overrightarrow{n}\cdot {\overline{\overline{\tau }}}^{\ast }\cdot {\overrightarrow{e}}_{x}]d{S}^{\ast },$$

where ${F}_{d}^{\ast }$ is the normalized drag force per unit length, $\mathop{n}\limits^{\rightharpoonup }$ is the surface normal vector, and ${\mathop{e}\limits^{\rightharpoonup }}_{x}$ is the x-direction unit vector. Due to the limited measurement area of the velocity field, our control volume is confined within a region close to the drop ($x/{D}_{p}\in [-0.84,\,1.425]$ and $y/{D}_{p}\in [-1.15,\,1.15]$) and exclude a significant portion of the streamers, which severely underestimates the drag as well as imposes large uncertainties in the calculated pressure, momentum flux, viscous forces and subsequently the drag on droplets with streamers. To assess uncertainties in the drag measurement, we estimate each mean drag using 25 control volumes with a fixed size maximally allowable for the analysis but with a varying centroid (details in *SI* S.2.2). A mean drag force (or drag coefficient, ${C}_{d}={F}_{d}/(0.5{\rho }_{f}{U}_{f}^{2}{D}_{d}^{2})$) is obtained by averaging estimations over these 25 fixed-size control volumes. The mean drag coefficients for each flow realization normalized by that of a smooth droplet (Fig. 4A) are presented in Fig. 5. The filled markers represent normalized drag coefficients for those instances when it has identified streamers, while open markers are for those without. Error bars are one standard deviation from the mean calculated from 25 control volume variations per time instance. Figure 5 clearly shows that streamers increase drag on the droplet, even with only a few of them. For instance, at Δ*t* = 30 min, the presence of two streamers causes ${C}_{d}/{C}_{d,0}$ to increase by 80% (Figs. 4B and 5), while 10 min later (Δ*t* = 40 min) the drag recovers immediately as they detach (Figs. 4C and 5). Another short moment later, drag increases by 120% when four streamers are formed (Fig. 4D and 5). Bearing in mind that the analysis only includes $\sim 1.5{D}_{d}$ downstream worth of a streamer that extends as long as >12*D*_*d*_ downstream (Fig. 1F), these drag estimates presented here are highly conservative. Such a streamer bundle is expected to cause a catastrophic reduction in droplets’ rising velocities and consequently may substantially alter the fate of oil droplets.

It is worth noting that the dramatic increase of drag by a single streamer is unexpected. Such a drastic increase in drag (>80%) cannot result from the frictional stress tangent to the streamer. Due to space limitation, we only discuss the mechanism qualitatively here and leave quantitative discussion to *SI* S.2 and Fig. S3. As shown in Fig. 4B, streamers do not follow streamlines such that apart from conventional shear stress tangent to the filament, additional viscous stresses normal to the filament owing to the crossflow cause the widening of streamlines and the development of a “wake”. Within this “wake”, the pressure behind the drop recovers slower than without streamers, which effectively increases the pressure difference before and after the drop with streamers, drastically increasing the drag, *a.k.a*. pressure drag. Evidence can be further drawn from the measurement at Δ*t* = 60 min (Fig. 5E) that although two streamers are present, drag close to that of a smooth droplet is obtained. Inspection of Fig. 4E reveals that in this instance the majority portion of streamers approximately follow streamlines that supports our assertion that normal stresses and pressure, not friction (or shear), is the true origin of unexpected larger drag than frictional drag. In short, streamers crossing the streamlines modify the pressure field behind the drop and drastically increase form drag that is normally absent in Stokes flow regimes.

### Implication of streamers on the potential fate of oil droplets

In our kernel experiment from Figs. 3–5, due to the formation of streamers, the drag increases rapidly (e.g. within 50 min in Fig. 5) and drastically (e.g. by more than 80% in 30 min in Fig. 5). A rough estimation (*SI* S.3) shows that an 80% increase in drag (Fig. 4B) will result in a 33% slower rising velocity of a 100 *μm* droplet; a 100% increase (Fig. 4G) causes a 50% drop; and a 10-times increase causes a 90% slower rising velocity. Note that due to the limitation in our flow measurement, we are unable to directly estimate the drag on a large aggregate that exceeds our field of view ($720\times 720\,\mu {\rm{m}}$). However, it is not unreasonable to expect a substantial increase (e.g. 10 times) in drag of a droplet with an elongated tail (e.g. >12 *D*_*d*_ in Fig. 1F) and an enlarged cross-section (e.g. >2.5 *D*_*d*_ in Fig. 2D). A 90% reduction in rising velocity for a 100 *μ*m droplet would yield an increase in its residence time in a 100 m layer from 3 to 35 d, thus allowing ample time for many biotic processes including biodegradation and MOS formed directly in the deep-sea plume. The short initiation time scale of a streamer compounded with its strong impact on droplet hydrodynamics particularly provides us with a plausible mechanism to support field observations of biodegradation and sedimentation^7,25–29^.

## Conclusion

**Figure 6:**
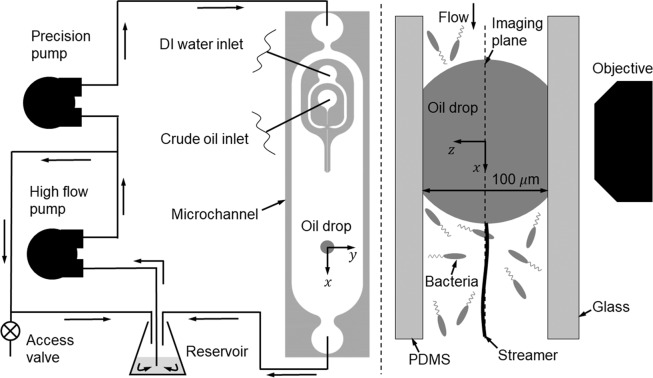
Schematics of the *Ecology-on-a-chip* (*eChip*) platform for mechanistic microcosm experiments. (Left) eChip platform with annotated components, (Right) Side-view of a pinned droplet in the microchannel viewed directly by a Nikon microscope (Nikon Ti-E) at the magnification of 20×.

We have provided clear experimental observations of microbial aggregates and streamers forming directly on a micro-scale oil droplet for the first time. We have demonstrated through our long-term microcosm experiments that the formation of EPS streamers and later larger aggregates can be a rapid and robust process, i.e. streamers can be initiated in less than 30 min after droplet’s exposure to a bacterial suspension. These aggregations change their morphology in stages such as cell attachment (Fig. 3A), streamer initiation and bundling (Fig. 3B,C), proliferation/growth (Fig. 3D), dispersal (Fig. 3E) and reformation (Fig. 3F), resembling the life of a biofilm over a solid substrate. Furthermore, these streamers can substantially increase drag as indicated by a control volume analysis of measured flow fields around a drop with and without attached streamers. For example, with only a few isolated streamers the drag on a drop is increased by at least 80% compared to a clean droplet (Fig. 6). Generally, a droplet experiencing increased drag would reduce rising velocities, enhancing residence times necessary for biotic processes and oil fates such as biodegradation and MOS formation.

While the phenomena described in this paper provides a practical pathway for biodegradation, MOS formation, and other biotic processes to occur directly in the water column, we stress that caution should be taken in making direct extrapolation with the real environment following the DWH explosion. Major differences between our laboratory experiments and the water column exist: (i) lack of microbial diversity, (ii) relatively high cell concentration (${10}^{8}\,{\rm{cells}}\,{{\rm{ml}}}^{-1}$ or $O{D}_{600}\approx 0.4$) in experiments to that in DWH plume (${10}^{6}-{10}^{7}\,{\rm{cells}}\,{{\rm{ml}}}^{-1}$) and (iii) elevated experimental temperature (20 °C) in comparison to that of the plume (4 °C). However, the results of this paper clearly demonstrate a previously unreported means for a bacterial suspension to significantly increase the residence time and essentially “trap” a rising sub-millimeter droplet in the water column. Without such known mechanisms it would be difficult to reconcile the competing time scales necessary for biodegradation and MOS formation as fates for sub-millimeter oil droplets following a deep sea oil spill. Thus, while further work in the context of *eChip* is necessary to more closely replicate the conditions of the deep sea, these results provide a crucial step in furthering our understanding of the fate of oil following a deep sea oil spill.

## Materials and Methods

### The Ecology-on-a-Chip (eChip) platform

More details can be found in White *et al*.^39^. The experimental setup consists of a chemostat/reservoir (150 ml flask), two peristaltic pumps, and a microfluidic channel (Figs. 6 and 3D rendering in Fig. 2 of ref. ^39^). These components are interconnected with soft 1/4” Tygon tubing (Cole-Parmer) while 1/16 in PEEK tubing (IDEX) is used to connect the microfluidic channel. Proper PEEK fittings (IDEX) connecting to PEEK tubing and polypropylene fittings (Cole-Parmer) connecting to Tygon tubing are selected to establish the close–loop microcosm environment.

There are two flow loops in the platform: a primary loop for continuous *in-situ* observations of microbe-oil interactions and a bypass loop to support *in-situ* microbial growth and monitoring. During each experiment after dispensing and pinning the drop in the microchannel, fluid drawn from the reservoir by a high flow peristaltic pump (INTLLAB) operated at a fixed 50 rpm with 1 mm inner diameter silicone tubing is split into two different loops at the T-junction. As a portion of the fluid enters into the main loop, the rest recirculates directly back to the reservoir via the bypass loop within which an access valve is integrated for removing or adding fluids. The fluid in the main loop is further pumped by an additional high precision micro-peristaltic pump (Masterflex C/L, Cole-Parmer) with Masterflex tubing. This micro-peristaltic pump provides exquisite flow control to regulate the flow rate in the microfluidic channel and eventually return to the reservoir. Note that periodic fluctuations inherent to peristaltic pumps still exist and may affect flow measurements around a droplet in the microchannel. It is found that at flow rates of about 150 *μ*l min^−1^(typical experimental flow rates) the flow regularly fluctuates at approximately 10 Hz. These fluctuations will not affect experiments and analysis based on mean flow if sufficient periods of flow fluctuation are captured and averaged.

The observation area in the microchannel, where a micro droplet is pinned, is imaged using a Nikon TiE transmission microscope with either 20X S Plan Fluor ELWD objective for differential interference (DIC) or 20X Plan Fluor DLL for phase contrast microscopy. The microscope is equipped with a large format EMCCD camera (iXon, Andor) for the long term (>days) time lapsed images and a high speed 1K × 1K CMOS camera for flow measurements. High speed images are recorded exclusively with the S Plan Fluor ELWD objective which has numerical aperture 0.45 and depth of field ~5 *μ*m. A newly developed microfluidic channel allows the generation of a single micro oil droplet with well-controlled size and the pinning of it at the observation area located in the open section of the microfluidic channel (as illustrated in Fig. 6). An oil droplet generation and dispersion sub-system is also developed using two individually controlled syringe pumps (New Era Pump) for dispersing oil and sterilized DI water as a buffer solution. The oil syringe is a chemically inert glass syringe (Hamilton Gastight, Fisher), while a 3 ml polyethylene syringe (BD) is used for the buffer.

### Microfluidic channel

**Figure 7:**
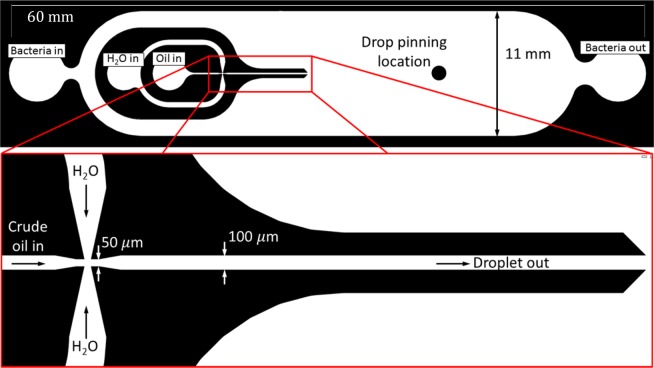
Layout of microchannel. Inset: flow focusing junction for the generation of a single oil droplet with a size of 100–600 *μm*.

The channel is capable of dispersing single oil droplet with well controlled droplet size. Figure 7 shows the mask schematics of the microchannel. The symmetric microchannel of $60\times 11\times 0.1\,{\rm{mm}}$, latter being the depth, has two primary fluid ports for the continuous fluid circuit connected to PEEK tubing, where bacterial suspensions can be driven into and out of the channel. A co-axial flow nozzle with flow focusing junction is designed to generate a single oil droplet with accurate size control. Shown in the inset of Fig. 7, the nozzle has two separate inlets: through the inner one the oil is injected to allow the generation of droplet, while through the outer one the buffer fluid (e.g. DI water) is injected to provide the oleophobic layers over the nozzle walls. The four-way junction with the narrowest cross-section opening of 50 *μ*m is used to create flow focusing to facilitate the pinch off of a single droplet with well controlled size. The dispensing protocol allowing single droplet generation must be well executed and is provided later.

The microfluidic channel is fabricated by soft lithography technique^52^ using poly(dimethylsiloxane) (PDMS) (Dow Corning). A chrome mask with the designed 2D microchannel and nozzle is generated using a Heidelburg mask writer. The use of a hard chrome mask instead of conventional soft film mask is necessary to produce a microscale flow focusing junction with sharp and straight side walls. The negative master of 100 *μ*m deep microchannel is created by using SU-8 photoresist and patterned by photolithography. To create the master, a 100 *μ*m layer of SU-8 negative photoresist (SU-8 2075, MicroChem) is spin-coated at 2200 rpm for 30 s over a 4 in Si-wafer, soft baked on a hotplate at 65 °C for 5 min first and followed by another soft bake at 95 °C for 20 min. The coated wafer is patterned by a Carl Suss mask aligner for 30 s using hard contact mode. The resist undergoes a post-exposure bake at 65 °C for 5 min and subsequently at 95 °C for 10 min. The master is developed in 1-methoxy-2-propanol acetate (Fisher) at room temperature for 17 min to fully reveal features of the microchannel. The baking protocols must be strictly followed to prevent thermal induced cracks often developed at the nozzle.

Microchannels are formed by molding PDMS over the master. PDMS is mixed at a ratio of 10:1 PDMS to cross-linking agent and degassed in a desiccator. The mixture is cast on the master and cured in an oven at 65 °C for 1 d. The cured PDMS mold is cut from the master, and holes for inlets/outlets are punched using a 1.5 mm biopsy punch. The PDMS channel is bonded to a glass slide pre-cleaned with “piranha” etch solution (99% H_2_SO_4_ and 30% H_2_O_2_ at 1:2 v/v) using air plasma activation for 1.5 min in a plasma cleaner (Harrick). A surface functionalization must be followed immediately after, since all inner surfaces of the channel made of glass and PDMS must be hydrophilic.

### Layer-By-Layer (LBL) surface functionalization

Crucial to the formation of isolated crude oil droplets in the microchannel is to maintain all contact surfaces as oleophobic. Note that PDMS is inherently hydrophobic and oleophilic due to its non-polar functional groups. Thus, oil naturally spreads on PDMS, making a droplet on-chip impossible. By forming a strongly hydrophilic surface, water would have a high enough affinity for the channel walls such that the water essentially blocks the crude oil from coming in contact with the channel surfaces, forming in effect an oleophobic surface.

Here we use a layer-by-layer deposition technique to form a polyelectrolyte layer on both PDMS and glass surfaces in the microchannel^53^. A brief summarization of the technique is as follows. Immediately after bonding the PDMS mold to the glass substrate using air plasma treatment, the channel is filled with 10 *μ*M poly(allylamine hydrochloride) (PAH). The PAH bonds to the charged channel walls and reverses the wall charge from negative to positive. After 5 min, the PAH is removed from the channel and rinsed thoroughly with 0.1 M NaCl buffer solution to remove remaining free PAH. Then the channel is filled with 10 *μ*M poly(sodium 4-styrenesulfonate) (PSS). The PSS bonds to the PAH layer, reversing the charge from positive back to negative. After 5 min, the PSS is removed and washed thoroughly with 0.1 M NaCl buffer solution to remove free PSS, and the channel is filled with PAH again. The washing step is very important due to free PAH and PSS easily forming salts, which contaminate the surface or even clog the channel.

The process is continued with alternating PAH/PSS depositions until the desired number of PAH-PSS layers are formed. Four layers of PAH-PSS are used in current experiments. Following the final deposition of PSS, the channel is rinsed thoroughly with DI water. Anecdotally, channels functionalized with the PAH-PSS coating are successfully used several months after manufacture, demonstrating the robustness of the technique. Additionally, a pinned crude oil drop in the functionalized microchannel can maintain both its pinned state and oleophobic contact angle with the PDMS and glass for at least 3 weeks of continuous flow, demonstrating the durability of the coating.

### Experimental conditions

The conditions for experiments discussed in the main text are summarized in Table 1. Each experiment is labelled in Column 1 of Table 1, and so are their corresponding main text figures (Column 2). The experimental conditions are organized into three categories: characteristics of oil phase, particle phase and flows used in each experiment. Note that the first experiment (E1 in Table 1) is abiotic.

### Culturing protocol of bacterial suspension

The biotic experiments in this study involving *Pseudomonas sp*. (strain P62, ATCC 27259) use a two-step growth protocol. The first growth is conducted in a flask on a rotary shaker. Then 20 ml of sterile Nutrient Broth ($8\,{\rm{g}}\,{{\rm{l}}}^{-1}$, Difco, BD Cat. No. 234000), is pipetted into a sterilized flask and inoculated with 100 *μ*l of −20 °C short term stock. The inoculated culture remains on a rotary shaker at 120 rpm and at room temperature (23 °C) until it reaches saturation growth (~4 days and *OD*_600_ > 1). This culture is used as the working stock for our microcosm experiments.

At the beginning of each microcosm *eChip* experiment after a crude oil droplet is dispensed and pinned in the microchannel, the reservoir is filled with 50 ml nutrient broth and the entire system is primed. With the high precision pump off 100 *μ*l of the working stock is inoculated through the access valve (Fig. 6). The high flow pump circulates the culture overnight through the bypass loop isolated from the microchannel to allow the culture to grow without interacting with the pinned droplet. When the culture in the reservoir reaches the lower mid-log growth (*OD*_600_ ≈ 0.4), the precision pump will be turned on to allow bacterial suspension to flow into the main loop and to interact with oil droplet. From herein on, the microcosm experiment starts.

### Preparation of particle suspensions with various purified EPSs

For comparative abiotic studies (E1–E4 in Table 1, Fig. 1A–D), 1 *μ*m polystyrene particles are used to mimic bacterial cells in these experiments. Before adding particles, the media (e.g. DI water in E1–2, 25 ppt NaCl in E3–4) are mixed with purified EPS extracted from either *Sagittula stellata* culture or microbial consortium from Gulf of Mexico (gifts from Santschi’s group, TAMUG) at the concentration equivalent to those in the water column. For details on the EPS extraction protocols please refer to^48^). The media is sterilized with a 0.2 *μ*m filter; this step is performed under a sterilized environment (e.g. a laminar hood with UV lamp) that is critical for preventing contamination. The sterile (i.e. no living bacteria present) EPS suspension flows through the microchannel containing the pinned droplet for 24 h, and then the polystyrene particles are introduced through the access valve. Daily examination of suspensions under a separate microscope (Nikon TS-100) ensures abiotic condition during microcosm experiments. In experiment E2 (Fig. 1B, Table 1), we show that accidental contamination causes the formation of streamers within one day after exposure to EPS-particle suspension. To showcase the impact of a small amount of live bacterial cells in EPS-particle suspension on the morphology of EPS aggregate, we conduct experiment E3 (Fig. 1C, Table 1) using the sterile EPS-particle suspension where the EPS is purified from *Sagittula stellata* culture^54^ and spiked with live *Sagittula* bacteria (*OD*_600_ ≪ 0.01), as well as the experiment E4 (Fig. 1D, Table 1) using only the sterile EPS-particle suspension (i.e. no bacteria) where the EPS is purified from indigenous consortia in Gulf of Mexico (GOM).

### Disperse and pin a droplet in microchannel

At the beginning of each microcosm experiment, a single oil droplet must be generated and pinned at the observation area in the microchannel. The single crude oil droplet is generated on-chip with a coaxial nozzle with a flow focusing junction (inset in Fig. 7) and two manually operated syringe pumps (New Era Pump). A Hamilton Gastight syringe is used for crude oil for precise dispensing. The second pump dispenses sterile DI water (buffer) from a sterile plastic syringe. With the high precision pump on and DI water syringe pumping at 10 $\mu {\rm{l}}\cdot {min}^{-1}$, the crude oil is injected at 100 ${\rm{nl}}\cdot {{\rm{\min }}}^{-1}$ (see Fig. 6 for schematic). In the microchannel the oil-carrying inner nozzle comes to the flow focusing junction with the water-carrying outer nozzle where a single crude oil droplet is pinched off (Video S5). Immediately after the pinch-off the crude oil syringe is turned off. The droplet then flows into the 11 mm wide channel until reaching the position in the observation area (Fig. 6). When droplet is attached at this position, the high precision pump and DI water syringe pump are turned off. The setup is left in this state overnight to (i) allow the droplet to be pinned to the top and bottom channel walls, and (ii) verify the apparatus and medium are sterile. Following the verification that the droplet is pinned and the setup is sterile, the experiment is ready to be inoculated with bacteria.

### Sterilization procedure

Sterilization is crucial for both our abiotic and biotic studies to properly interpret the experimental results. All tubing, fittings (except the access valve), the reservoir flask, silicone stopper and syringes are autoclaved at 121 °C for 30 min. Non autoclavable components including the microchannel and access valve are washed thoroughly with 70% ethyl alcohol for sterilization for at least 30 min. Following sterilization, the tubing circuit is assembled in a laminar flow hood with UV and 50 ml of sterile medium is added to the reservoir flask. These components are then carefully setup on the Nikon Ti-E microscope according to the schematic in Fig. 6. Careful pre-check procedures (discussed above) are strictly followed to ensure the entire microcosm setup is sterile before introducing bacteria.

### Image acquisition

A Nikon Ti-E microscope at 20X magnification - Nikon Plan Fluor DLL (for phase contrast) or S Plan Fluor ELWD (for differential interference contrast or DIC) - is used to provide both time-lapsed observations and flow measurements during experiments often lasting for days. Two image streams, e.g. long term time lapsed images to monitor the morphology change of droplet and time evolution of flow fields around it, are acquired concurrently by two different cameras with proper synchronization. With an $1K\times 1K$ EMCCD camera (Andor), time lapsed images are acquired every 30 s for the duration of each experiment, and streamed directly to a data storage. Concurrently, with an IDT high speed $1K\times 1K$ CMOS camera, a series of high speed image recordings are made at an interval of 10 min. Each high speed acquisition composed of 1000 images is recorded at 1000 fps for 1 s to the on-camera memory and automatically downloaded to data storage after each acquisition. Using a custom automation Matlab script, the microscope automatically switches back and forth synchronously between the camera port of EMCCD and that of CMOS camera. Both cameras are automatically triggered internally to capture both image streams continuously, i.e. one stream records images of oil water interface every 30 s, while the other provides flow measurements every 10 min, which allows the experiment to run unattended for days. The experiments are data intensive, e.g. a 5 d experiment results in 500 GB raw images.

### Measurement of mean flow around a drop

To obtain time evolution of flow fields around an oil droplet in the microfluidic channel and the subsequent estimation of drag, micro Particle Image Velocimetry (μPIV) technique is implemented with a Nikon Ti-E transmission microscope and a large format high speed camera (IDT-NR4). The flow measurement area is $720\times 720\,\mu {\rm{m}}$ covering the entire drop with the magnification of 20×. Note that since a 20X Nikon S Plan Fluor ELWD objective (numerical aperture = 0.45) has the depth of field (DOF) of 5 *μ*m. instantaneous flow measurements are averaged over a depth of 5 *μ*m. We need to emphasize that the image plane is placed squarely at the center of the channel far away from all channel walls (approximately 45 *μ*m from both the top and bottom wall). Each mean velocity field is measured at an interval of 10 min for the duration of each microcosm experiment.

Due to intrinsic fluctuations generated by the peristaltic pump, a regular periodic fluctuation at ~10 Hz is measured in the velocities. Each mean flow field at a given time is the direct result of ensemble averaging over 999 instantaneous velocity maps obtained from a sequence of high speed recording over 1 second period at the rate of 1000 fps. This one second recording period is short enough to “freeze” the flow at any given sampling time, but long enough to capture sufficient periods of flow fluctuations generated by the peristaltic pump. The calibration measurement of velocity in the same microchannel using a particle suspension at the same flow rate (148 ${\rm{u}}{\rm{l}}\cdot {{\rm{m}}{\rm{i}}{\rm{n}}}^{-1}$) as in experiment E7 (Table 1, Figs. 3–5) shows that within 1 second we have captured >9 periods of flow fluctuation. We have performed ensemble averaging over the entire 1 second period and compared the mean field to that averaged over exactly 9 periods of flow fluctuation. It is shown that the mean error introduced is 0.3%. Thus we opt to simply average over the entire 1 s period per sequence indiscriminately without risk of introducing significant errors.

In our experiments, we use bacteria cells as tracer particles for flow measurement. The justification is two-fold: *Peclet* number for the bacteria is much larger than 1 suggesting that bacterial cells act like solid passive particle and their swimming motility has only negligible influence on flow measurement; and Stokes number ($Stk=2/9({\rho }_{b}/{\rho }_{f}){({d}_{b}/{D}_{d})}^{2}R{e}_{D}$, where *ρ*_*b*_ is the bacterial cell density, *d*_*b*_ is the characteristic size of bacterium, *D*_*d*_ is drop diameter, and *Re*_*D*_ is Reynolds based on drop diameter) are on the order of 10^−5^ that indicates bacteria cells behaving as solid particles will follow the flow streamlines. Since *μ* PIV techniques have been widely used in literatures, we will only briefly summarize the procedures used to obtain the mean velocity field for each high speed sequence:

(i) After the acquisition of each 1 s high speed sequence containing 1000 images spaced 1 ms apart in time, conventional cross-correlation based PIV analysis^50,55^ is applied to every two consecutive images in the sequence resulting in a total of 999 velocity maps. Note that the density of bacteria cells are sufficiently high in our experiments to adequately resolve flow around a drop with 48 by 48 pixel windows at 16 pixel increments in the *x-* and *y-*directions.
(ii) Once an instantaneous velocity field per an image pair is calculated, a PIV-assisted Particle Tracking Velocimetry [PTV^50^], is applied to these cell locations extracted from the image pair to obtain individual cell displacements. We can obtain ~3000 individual velocity vectors per image pair. To highlight the ability to obtain highly resolved instantaneous velocity measurements, we superimposed five randomly selected velocity maps out of 999 in a high speed sequence in Fig. 3A. It is clear that our single instantaneous measurement resolves the flow field around a drop with sufficient resolution, even in close proximity to the oil water interface. Each of unstructured PTV velocity maps is then interpolated onto structured grids with a resolution of 2.7 *μ*m (or 4 pixels) in both *x-* and *y-*directions.
(iii) The mean velocity field for this image sequence is averaged over 999 instantaneous realizations. We need to emphasize that due to intrinsic fluctuation from the pump, a faithful estimation of the mean flow field at given time must be computed over a portion of each sequence covering sufficient fluctuations. As discussed above, the error introduced by averaging non-integer number of periods of flow fluctuation is only 0.3%. To expedite the processing of a large amount of data, we estimate the mean field using the entire 1 s sequence.

## Supplementary information

Video S1.

Video S2.

Video S3.

Video S4.

Video S5.

Supplemental Materials.
