# Supplementary material for: Bacteria forming drag-increasing streamers on a drop implicates complementary fates of rising deep-sea oil droplets
Source: Sci Rep. 2020 Mar 9;10:4305. doi: 10.1038/s41598-020-61214-9 (PMC7062730; doi:10.1038/s41598-020-61214-9)
Supplement: Supplementary file 6 — Supplemental Materials. [file 41598_2020_61214_MOESM6_ESM.pdf]

Supplemental Materials:

Bacteria forming drag-increasing streamers on a drop implicates complementary fates of rising deep-sea oil droplets

Andrew R. White[†], Maryam Jalali[†], Michel C. Boufadel[‡], Jian Sheng[†*]

[†]Dept. of Engineering, Texas A&M University–Corpus Christi, Corpus Christi, TX 78412, USA

[‡]Center for Natural Resources, Dept. of Civil and Environmental Engineering, New Jersey Institute of Technology, Newark, NJ 07102, USA

*Corresponding author: Jian Sheng, Dept. of Engineering, Texas A&M University–Corpus Christi, 6300 Ocean Dr., Corpus Christi, TX 78412, Tel: (361)825-3731. Email: jian.sheng@tamucc.edu

S.1 Characteristics of model bacterium, *Pseudomonas* sp. (P62): motility, dispersion, and chemotaxis

Translational motility and dispersion. To measure dispersion and motility of *Pseudomonas* P62, cultures are grown in nutrient broth (Difco BD Cat. No. 234000) with varying additional salinities of 0, 10 and 25 parts per thousand (ppt). Cultures are gown on a rotary shaker at 100 rpm and room temperature. Cells were then washed and re-suspended in various media (Table S1) to measure the swimming motility of *Pseudomonas* (P62) and its effective dispersions. Bacteria trajectories in the quiescent fluid within the microchannel were measured using digital holographic microscopy (DHM) and in-house software [1]. These trajectories are used to measure linear and angular velocity, and dispersion rate. The dispersion coefficients are measured using ensemble averaging of the velocity autocorrelation function, $R_{ii}(\tau) = u_i(t)u_i(t+\tau)$, determined for individual bacteria trajectory

$$D_{ii}(t) = \int_0^t R_{ii}(\tau)d\tau = \int_{\tau=0}^{t} \int_{\eta=0}^{\infty} u_i(\eta)u_i(\eta+\tau)\, d\eta d\tau \qquad \text{(S1)}$$

where τ is time, u is the fluctuation component of bacteria swimming velocity, and the subscript i refers to a direction, x, y or z. Due to the limited spatial extent of essentially all velocity measurement systems, including DHM, the autocorrelation function can be measured only over a finite time. Consequently, Snyder and Lumly [2] have introduced the idea of ensemble averaging of many particle trajectories, each with a finite length. The revised diffusion coefficient, $D_{ii}(t)$, is thus determined from:

$$D_{ii}(t) = \langle \int_0^t d\tau \int_0^\tau R_{ii}(\eta) d\eta \rangle = \int_0^t d\tau \int_0^\tau \langle R_{ii}(\eta) \rangle d\eta \quad \text{(S2)}$$

where $\langle \quad \rangle$ denotes ensemble averaging performed over trajectories of the same species. The asymptotic value of $D_{ii}(\tau)$, as $\tau \to \infty$, yields the Fickian diffusion coefficient.

Chemotaxis. *Pseudomonas* P62 chemotaxis (or lack thereof) near a crude oil-water was studied using a straight 200 μm deep and 1 cm wide microchannel wherein half of the channel (500 μm thick) is crude oil and the other is bacteria culture. This produces a quiescent rectilinear oil-water interface at which DHM is used to measure bacterial swimming trajectories. Figure S2 shows the probability density functions (PDF) of swimming velocities within 300 μm of the oil-water interface. Results at 0, 1 and 4 h after exposure to the oil interface are shown. The data indicate that the mean velocities in the culture near the crude oil show no bias towards or away from the interface. Therefore, we conclude that *Pseudomonas* P62 exhibits no chemotactic behaviors towards crude oil.

S.2 Analysis of flow and hydrodynamic drag

S.2.1 Estimation of momentum budgets.

To estimate the drag on the droplet, we estimate the 2D momentum balance around a droplet. The 2-D momentum balance at the imaging plane (the mid-plane) of the microfluidic channel can be expressed as

$$\frac{\partial \vec{u}}{\partial t} + (\vec{u} \cdot \vec{\nabla})\vec{u} = -\frac{1}{\rho_f}\vec{\nabla} p + \nu_f \vec{\nabla} \cdot \vec{\nabla} \vec{u}, \quad \text{(S3)}$$

where $\vec{u}$ is mean flow velocity, $\vec{\nabla}$ is the gradient operator, ρ_f and ν_f are the density and kinematic viscosity of the surrounding fluids respectively. For the flow around a micro droplet with Re << 1 and a steady rising velocity, the unsteady term (1st term) n Eq. S1 can be neglected. The steady momentum balance can be expressed as the following:

$$u_x \frac{\partial u_x}{\partial x} + u_y \frac{\partial u_x}{\partial y} = -\frac{1}{\rho}\frac{\partial p}{\partial x} + \nu \left(\frac{\partial^2 u_x}{\partial x^2} + \frac{\partial^2 u_x}{\partial y^2} \right)$$

$$u_x \frac{\partial u_y}{\partial x} + u_y \frac{\partial u_y}{\partial y} = -\frac{1}{\rho}\frac{\partial p}{\partial y} + \nu \left(\frac{\partial^2 u_y}{\partial x^2} + \frac{\partial^2 u_y}{\partial y^2} \right) \quad \text{(S4)}$$

where u_x and u_y are *x*- (streamwise) and *y*- (spanwise) components of mean fluid velocity around the droplet. To scale our results later to other droplet size, we express the momentum equation in their dimensionless forms:

$$u_x^* \frac{\partial u_x^*}{\partial x^*} + u_y^* \frac{\partial u_x^*}{\partial y^*} = -\frac{1}{Re_D}\frac{\partial p^*}{\partial x^*} + \frac{1}{Re_D} \left(\frac{\partial^2 u_x^*}{\partial x^{*2}} + \frac{\partial^2 u_x^*}{\partial y^{*2}} \right),$$

$$u_x^* \frac{\partial u_y^*}{\partial x^*} + u_y^* \frac{\partial u_y^*}{\partial y^*} = -\frac{1}{Re_D}\frac{\partial p^*}{\partial y^*} + \frac{1}{Re_D}\left(\frac{\partial^2 u_y^*}{\partial x^{*2}} + \frac{\partial^2 u_y^*}{\partial y^{*2}}\right). \quad \text{(S5)}$$

All momentum budgets listed in Eqn. S5 can be estimated faithfully owing to our highly resolved mean velocity fields (e.g. at spatial resolutions of 2.7 μm) except for the pressure gradient terms. By balancing the momentum in both *x*- and *y*-axis, one can obtain the elusive pressure gradient and subsequently pressure distributions around a droplet:

$$\frac{\partial P^*}{\partial x^*} = \frac{\partial^2 u_x^*}{\partial x^{*2}} + \frac{\partial^2 u_x^*}{\partial y^{*2}} - Re_D \cdot \left(u_x^* \frac{\partial u_x^*}{\partial x^*} + u_y^* \frac{\partial u_x^*}{\partial y}\right),$$

$$\frac{\partial P^*}{\partial y^*} = \frac{\partial^2 u_y^*}{\partial x^{*2}} + \frac{\partial^2 u_y^*}{\partial y^{*2}} - Re_D \cdot \left(u_x^* \frac{\partial u_y^*}{\partial x^*} + u_y^* \frac{\partial u_y^*}{\partial y}\right) \quad \text{(S6)}$$

It is worth to point out that the relative pressure field can be obtained by integrating the above pressure gradient fields: $\partial p^*/\partial x^*$ and $\partial p^*/\partial y^*$, but the absolute pressure depends on pressure along the boundary. The magnitude of pressure gradients, $\sqrt{(\partial p^*/\partial x^*)^2 + (\partial p^*/\partial y^*)^2}$, for the first 100 minutes in our kernel experiment (E7 in Table 1, Figs. 3-5 in main text) are plotted in Fig. 5, showing shapes and locations of the streamers trailing the droplet. Figure S3 further substantiates our assertion in the main text that a single streamer is too thin and transparent to be visible in the recorded micrograph and can only be identified by the attached cells along the streamer or by superimposed pressure gradients. Shown in Fig. S2B, two streamers can be identified but only by attached bacterial clusters marked by arrows (solid: streamer 1; hollow: streamer 2). For a video of this high speed sequence see Video S5. Figure S3C shows the contours of pressure gradient magnitude contours over the micrograph. It is clear that regions with elevated pressure gradient magnitude indicate unequivocally the locations of those two streamers.

S.2.2 Control volume analysis for estimating drag over a droplet

To estimate the drag force on the droplet from mean velocity measurement, a control volume analysis over *x*-momentum is performed. A control volume is drawn around the periphery of the velocity field as shown in Fig. S3A. In this 2D schematic, the stationary drop (gray circle) is enclosed by a control region ABCD (Fig. S3A) with *x*-, *y*-axis and normal vector, $\vec{n} = n_x \overrightarrow{e_x} + n_y \overrightarrow{e_y}$ where $\vec{e}_{x,y}$ is the unit direction vectors for *x*- and *y*-axis. Using the control volume defined in Fig. S3A, the dimensionless steady *x*-momentum balance across the control region is (for brevity, Einstein index notation is used hereinafter):

$$\int_{ABCD} \left[Re_D\, (\vec{n} \cdot \vec{u}^*) u_x^* + n_x p^* - \vec{n} \cdot \vec{\vec{\tau}} \cdot \vec{e}_x\right] dS^* + F_d^* = 0, \quad \text{(S7)}$$

where "·" denotes dot product operator, $\vec{\vec{\tau}}^*$ is a dimensionless viscous stress tensor defined by $\vec{\vec{\tau}}^* = \vec{\nabla}^*\vec{u}^* + \left(\vec{\nabla}^*\vec{u}^*\right)^T$, dS^* is area of the local control surface and F_d^* the total dimensionless drag force on the drop per unit length into the paper. Evaluating every term in Eqn. S7 except for drag over the control surface, and integrating over it, the total drag force can be expressed as

$$F_d^* = \int_{A,C}\left[Re_D\left(u_{x,A}^{*2} - u_{x,C}^{*2}\right) + \left(p_A^* - p_C^*\right) - \left(\tau_{xx,A}^* - \tau_{xx,C}^*\right)\right] dy^* + \cdots$$

$$\int_{B,D}\left[Re_D\left(u_{y,D}^* u_{x,D}^* - u_{y,B}^* u_{x,B}^*\right) - \left(\tau_{yx,D}^* - \tau_{yx,B}^*\right)\right] dx^*. \quad \text{(S8)}$$

Although the pressure gradients are well resolved, absolute pressure distribution over the entire control volume is difficult to determine and depends on pressure on the boundary. However, since the relative pressure is only needed along the boundary, pressure gradients can then be integrated along the boundary, ABCD. Integration is performed using first order forward/backward finite differences. From two corners of the control volume, the resolved pressure gradient field is integrated in both clockwise and counterclockwise direction along ABCD. The resulting pressure profiles from both directions beginning at both points are averaged to produce pressure distributions along boundary A and C, p_A^* and p_C^*, where the subscript "A" and "C" describe the specific boundary. Examples of the velocity, pressure and stress profiles (i.e. momentum balance "budgets") over the control volume are shown in Fig. S3B-F. Shown here are results for x momentum budget distributions from mean flow measured at $\Delta t = 20$ min (no streamers, Figs. 3B, 4A, blue in Fig. S3), 30 min (with two streamers, Figs. 4C, 5B, red in Fig. S3), and 40 min (no streamers, Fig. 5C, green in Fig. S3). Budgets estimated at 30 min deviates substantially from those computed at the instances of a smooth droplet.

Analysis of the magnitudes of the budgets illustrates that streamer-induced changes in normal viscous stress (τ_{xx}^*) and pressure (P^*) are the dominant factors in increasing F_d^*, while changes in momentum and shear stress are an order of magnitude lower (Fig. S3B, E and F). Hence, we conclude with anecdotal evidence that *form drag* (i.e. pressure drag) rather than *friction drag* contributes substantially to the increase of the droplet drag. Form drag is not characteristic of the Stokes flow regime, however the realization that flow streamlines cross the streamers (Fig. 5B) can explain its existence. In the Stokes regime, and for very thin filaments such as the initial streamers with sparsely attached bacteria, the tangential and normal forces acting on the streamer are approximately proportional to the tangential and normal velocity components according to resistive force theory [3], i.e.

$$\boldsymbol{F}_{\parallel} = 4\pi\mu\varepsilon_{\parallel}U_{f,\parallel}^{r}l, \text{ and} \quad \text{(S9)}$$

$$\boldsymbol{F}_{\perp} = 4\pi\mu\varepsilon_{\perp}U_{f,\perp}^{r}l \quad \text{(S10)}$$

where l is the length of a filament segment, $\varepsilon_{\perp} = [\ln{(2l/b]}^{-1}$ with b being the filament diameter, and $\varepsilon_{\perp}/\varepsilon_{\parallel} \approx 2$. Fig. S3A shows schematically how the flow velocity components as well as the resultant forces are decomposed on the streamer. The presence of a normal force on the streamer causes the flow to deflect (Fig. 4D), forming anti-symmetry between the front and back of the droplet and importantly producing significant deviations in the pressure which leads to form drag.

The momentum deficit, viscous stress and pressure profiles are integrated over the control volume boundary ABCD using linear quadrature to obtain F_d^* (Eqn. S8). Note that our control volume has very limited size that includes only a small portion of an elongated streamer. This limitation will first severely underestimate the estimated drag on the drop and subject the sensitive integration of Eqn. S8 to the selections of control volume as well as integration methods. To circumvent this limitation, the integrations are performed several times over a control volume with fixed size but varying centroid, and the drag for each control volume is estimated individually. A discussion of how the control volume is chosen and varied follows.

Note that the control region is bounded by x_1^* (top), x_2^* (bottom), y_1^* (left) and y_2^* (right) in Fig. S3A. Due to inherent flow measurement errors near the border of the flow field, the control volume is only ever allowed to enclose grid points that are greater than or equal to 10 grid points normal to the edge of the flow field. The first control volume is defined such that x_1^* and y_1^* are 10 grid points from the top and left border, respectively, and x_2^* and y_2^* are 14 grid points from the bottom and right border, respectively. This allows the control volume size to be fixed while the centroid is shifted over 5 grid points in the x- and y-directions without violating the rule that the control volume does not include points within 10 grid points of any border. Thus, the drag is individually estimated from 25 different control volumes of fixed size and then averaged to get the mean drag. The drag force is reported using the drag coefficient C_d. Due to the viscous scaling of F_d^*, the resultant drag coefficient used here is

$$C_d = \frac{2\mu F_d^*}{\rho UD} = \frac{2F_d^*}{Re}. \quad \text{(S11)}$$

The mean and standard deviation of C_d are plotted as symbols and error bars in Fig. 6.

S.3 Discussion on the implications of increased drag on the fate of the deep-sea plume

S.3.1 Rising velocity of a crude oil droplet without and with trailing streamers

Owing to the density of crude oil (taken as $\rho_d = 900\ \mathrm{kg\ m^{-3}}$) being less than water, a spherical crude oil droplet will rise due to buoyancy in the water column. The equation of motion of a drop rising through a fluid is described as:

$$\left(\rho_d + \frac{1}{2}\rho_f\right) V_d \frac{dU_d}{dt} = \left(\rho_d - \rho_f\right) g V_d - F_d, \tag{S12}$$

where $V_d = \frac{1}{6}\pi D_d^3$ is the volume of the drop and F_d is the drag force on the drop. The left-hand-side (LHS) term is the rate of change of momentum including the added mass, $\frac{1}{2}\rho_f V_d$, due to acceleration of the drop; and the right hand side (RHS) of Eqn. S10 contains the buoyancy (1st term) and drag (2nd term). As the drop reaches its terminal velocity, the LHS vanishes and $F_d = \frac{1}{6}\pi D_d^3 \rho_f (SG - 1) g$. We make the reasonable assumption that the droplet rises approximately like a solid sphere. For a solid sphere rising with $Re_D \lesssim 0.5$ the drag will follow closely to Stokes drag law $F_d = 3\pi\mu_f U_d D_d$ and its terminal velocity becomes

$$U_d = \rho_f (SG - 1) g D_d^2 / 18\mu_f \tag{S13}$$

where ρ_f is the density of the surrounding fluids, $SG = \rho_d/\rho_f$ is the specific gravity of the drop to the surrounding fluids, D_d is the drop diameter and $\mu_f = 1.5$ and $1.08\ \mathrm{mPa \cdot s}$ are the water viscosity at 4 °C and 20 °C, respectively.

Using terminal velocities predicted by Eqn. S11 one can predict how long it would take a drop to rise 100 m (approximate thickness of a microbial bloom in the deep-sea plume). For example, a 100 μm droplet would rise according to Stokes drag at approximately $U_d = 0.36\ \mathrm{mm\ s^{-1}}$ at 5 °C, and the time it would take said drop to rise 100 m would be 3.2 d at 5 °C. In the context of typical environmental processes, this is a very short time. For instance, Hu *et al.* [4] has demonstrated the half-lives, $T_{1/2}$, of *n*-alkanes contained in suspended 10 μm oil droplets due to bacteria present in the deep-sea plume are 6-8 d with an initial lag, T_{lag}, of 5-10 d. For a 100 μm droplet rising through the microbial bloom of 100 m, it would have to rise less than half as fast as its natural rising velocity to overcome the initial lag period to allow biodegradation processes to have a chance to affect *n*-alkane distribution in the plume.

To compare the velocities between a clean drop and a drop with increased drag due to attachment of microbial streamers, we express the hydrodynamic drag of a drop coefficient C_d depends on drop surface properties, i.e. with or without streamer, and varies with time and rising velocity. For a time-invariant drag, the terminal rising velocity can be approximated as $U_d =$

$1.1547 C_d^{-0.5}(SG-1)^{0.5} g^{0.5} D_d^{0.5}$. In Fig. S4 plots of the ratio of terminal velocity for a drop with streamers to that of a clean drop, $U_p/U_{p,0}$, is plotted versus the ratio of drag coefficient for a drop with streamers to that of a clean drop, $C_d/C_{d,0}$. For drops with $D_d < 250\ \mu$m the relationship between $U_p/U_{p,0}$ and $C_d/C_{d,0}$ collapses onto a single curve owing to the fact that here drag coefficients follow the Stokes drag law where $C_d = 24/Re_D$.

For Stokes flow regime, a 60% increase in drag would result in a rising velocity approximately 33% slower; a 100% increase in drag would result in a rising velocity approximately 50% slower; and a 10-fold increase in C_d would result in U_d approximately 90% slower. A 90% reduction in rising velocity for a 100 μm drop at 5 °C would result in an increase in residence time in a 100 m span of the water column from 3 (< T_{lag}) to 35 d ($\approx T_{lag} + 3T_{1/2}$), thus allowing ample time for biodegradation processes. Note that the assumption on time-invariant drag coefficient is reasonable, since the relaxation time scale for a drop to reach its terminal velocity is around milliseconds for a micro drop and that for generating streamers is in minutes.

It should be stressed here again that the drag coefficient increases measured in this manuscript are considerably conservative due to our limited viewing area not capturing the full scope of the streamers. Furthermore, the drag coefficient increases reported here are occurring within the first two hours of exposure to bacteria, and as Figs. 1 & 2 demonstrate, the bacterial aggregates grow considerably larger afterwards. Undoubtedly these very large aggregates and bundled streamer tails will result in considerable increases in drag. Additionally, the microcosm apparatus used in this study considers only a fixed velocity. In a real system, as the streamer structures develop and form large drag-inducing tails, the drop velocity would slow down, accelerating the accumulation of additional bacteria and creating a "snow-balling" effect where larger and larger aggregates are formed. Therefore, this process becomes a positive feedback loop wherein bacteria aggregate, the drop slows down, bacteria aggregation accelerates, and the drop slows down further. When enough dense mass has accumulated on the droplet, or as one bacteria-drop aggregate collides with another, it will become neutrally or even negatively buoyant.

S.3.2 Implications of increased residence times of oil droplets

Streamers by bacteria on a rising oil droplet will reduce its velocity and subsequently increase its residence time in the deep-sea plume. As a result processes such as Marine Oil Snow (MOS) formation can readily occur *in situ* in the deep-sea plume where a microbial bloom has been reported [5-15]. This MOS can trap oil in the plume region for extended times leading to enhanced

biodegradation as well as cause the oil to sink to the sea floor as sediment which is corroborated by field data [16-21]. If oil droplets from the deep-sea plume are the source of sedimenting oil and are subject to enhanced degradation, it would be expected that indicators of biodegradation such as *n*-C_{17}:pristane ratios [22] should indicate enhanced degradation *below* the plume.

To test this hypothesis we use the BP Gulf Science Data inventory of water chemistry data from the Natural Resource Damage Assessment (NRDA) and Response (file WaterChemistry_W-01v02-01.csv). The extensive database was filtered with the following criteria. Only samples taken between May 11, 2010 and June 3, 2010 (last day before start of partial capture with Top Hat #4) were considered for this quick analysis. Then only samples with *both* measurable *n*-heptadecane (*n*-C_{17}) and pristane (Pr) were collected. The *n*-C_{17} : Pr ratios and their x-y-z coordinates were then readily obtained to determine their depth and distance from the wellhead (longitude -88.3667, latitude 28.7396). The ratios are compared to the reported Macondo reservoir *n*-C_{17}:Pr ratio which is 1.71 [23]. Then, the fraction of *n*-C_{17} apparently degraded relative to the reference Macondo reservoir is determined by

$$\phi = 1 - \frac{\left[\frac{\mathrm{C_{17}}}{\mathrm{Pr}}\right]_{\mathrm{WC}}}{\left[\frac{\mathrm{C_{17}}}{\mathrm{Pr}}\right]_{\mathrm{reservoir}}}, \qquad \text{(S14)}$$

where the subscript "WC" stands for "water column". For conciseness, we use the symbol ϕ to represent the relative degree of degradation.

Plots of ϕ are shown in Fig. S5 versus both depth below the sea surface (vertical axis) and distance from the wellhead (horizontal axis). Magnitudes of ϕ are indicated by the colors according to the color bar, and markers size increases with increasing ϕ for clarity. Assuming degradation processes occur at roughly the same time scales in and around the plume, these ϕ values essentially indicate how long droplets have resided at varying depth and distance from the wellhead. Several key observation can then be made from the plot in Fig. S5. A clear clustering of relatively low ($\phi < 0.3$) degradation is seen between depths of 1000-1300 m (between the dashed lines in Fig. S5) and extending up to 15 km from the wellhead i.e. within the deep-sea plume. These low ϕ values suggest plume droplets have resided in the water column for relatively short times. Less than 5 km from the wellhead, larger degradation ($\phi > 0.3$) is seen both above and below the plume. Here droplet transport may be from the plume upwards, from the plume downwards, and/or from the surface oil slicks downwards. However, >5 km from the wellhead, larger ϕ are primarily within or below the plume (shaded oval in Fig. S5). This suggests droplet

transport is primarily from the plume downward i.e. plume droplets >5 km from the wellhead underwent enhanced degradation and sedimentation. This anecdotal evidence from the field data provides additional plausibility for the mechanism of bacteria forming drag-increasing streamers directly on oil droplets in the deep-sea plume in the aftermath of the *Deepwater Horizon* oil spill, causing them to drastically increase their residence times in the water column and even sink to the sea floor as sediment.

Video Legends

Video S1. A video of the oil drop from Fig. 2 (Table 1) just after pinning is shown. This demonstrates that the drop is pinned to the top and bottom channel wall, but the liquid-liquid interfaces remain highly mobile. The flow is from top to bottom in the video and the scale bar is 100 μm.

Video S2. A video demonstrating a streamer attached to a droplet upstream (upstream to current frame) growing downstream and attaching to another droplet to form a larger aggregate. The experiment shown in this video is the same shown in Fig. 1G.

Video S3. A complete time lapse video of the kernel experiment using *Pseudomonas* corresponding to Fig. 2 is shown. The scale bar is 100 μm and the time since initial exposure to the bacteria is shown in the top left.

Video S4. A sample high speed sequence recorded at the rate of 1000 fps for the experiment shown in Fig. 3-5 to assess streamers' hydrodynamic impact is shown at $\Delta t = 30$ min after initial exposure to bacteria (Fig. 3C and 4B). Playback is 30X slowed down. Three green arrows on the left side of the rear of the drop indicate bacterial clusters trapped in a single streamer. A second streamer (not indicated) can be observed to the right of the indicated streamer.

Video S5. A video of a single crude oil droplet (dark fluid) being pinched off on-chip in the geometry from Fig. 7 is shown. The channel width is 100 μm and the constriction at the junction is 50 μm wide.

Reference:

1 Molaei, M. & Sheng, J. Succeed escape: Flow shear promotes tumbling of Escherichia colinear a solid surface. *Sci Rep* **6**, 35290 (2016).

2 Snyder, W. H. & Lumley, J. L. Some measurement of fluid-particle motion in an isotropic turbulent field. *J Fluid Mech* **48**, 41-71 (1971).

3 Gray, J. & Hancock, G. The propulsion of sea-urchin spermatozoa. *J. Exp Biol* **32**, 802-814 (1955).

4 Hu, P. *et al.* Simulation of Deepwater Horizon oil plume reveals substrate specialization within a complex community of hydrocarbon degraders. *Proc Natl Acad Sci USA* **114**, 7432-7437 (2017).

5 Valentine, D. L. *et al.* Propane respiration jump-starts microbial response to a deep oil spill. *Science* **330**, 208-211 (2010).

6 Kessler, J. D. *et al.* A persistent oxygen anomaly reveals the fate of spilled methane in the deep Gulf of Mexico. *Science* **331**, 312-315 (2011).

7 Camilli, R. *et al.* Tracking hydrocarbon plume transport and biodegradation at Deepwater Horizon. *Science* **330**, 201-204 (2010).

8 Joye, S. B., MacDonald, I. R., Leifer, I. & Asper, V. Magnitude and oxidation potential of hydrocarbon gases released from the BP oil well blowout. *Nat Geos* **4**, 160-164 (2011).

9 Hazen, T. C. *et al.* Deep-sea oil plume enriches indigenous oil-degrading bacteria. *Science* **330**, 204-208 (2010).

10 Du, M. & Kessler, J. D. Assessment of the spatial and temporal variability of bulk hydrocarbon respiration following the Deepwater Horizon oil spill. *Environ Sci Technol* **46**, 10499-10507 (2012).

11 Redmond, M. C. & Valentine, D. L. Natural gas and temperature structured a microbial community response to the Deepwater Horizon oil spill. *Proc Natl Acad Sci USA* **109**, 20292-20297 (2012).

12 Mason, O. U. *et al.* Metagenome, metatranscriptome and single-cell sequencing reveal microbial response to Deepwater Horizon oil spill. *ISME J* **6**, 1715-1727 (2012).

13 Dubinsky, E. A. *et al.* Succession of hydrocarbon-degrading bacteria in the aftermath of the Deepwater Horizon oil spill in the Gulf of Mexico. *Environ Sci Technol* **47**, 10860-10867 (2013).

14 Kleindienst, S. *et al.* Diverse, rare microbial taxa responded to the Deepwater Horizon deep-sea hydrocarbon plume. *ISME J* **10**, 400-415 (2016).

15 Valentine, D. L. *et al.* Dynamic autoinoculation and the microbial ecology of a deep water hydrocarbon irruption. *Proc Natl Acad Sci USA* **109**, 20286-20291 (2012).

16 Chanton, J. *et al.* Using natural abundance radiocarbon to trace the flux of petrocarbon to the seafloor following the Deepwater Horizon oil spill. *Environ Sci Technol* **49**, 847-854 (2015).

17 Yan, B. *et al.* Sustained deposition of contaminants from the Deepwater Horizon spill. *Proc Natl Acad Sci USA* **113**, E3332-E3340 (2016).

18 Stout, S. A. *et al.* Assessing the footprint and volume of oil deposited in deep-sea sediments following the Deepwater Horizon oil spill. *Marine Poll Bul* **114**, 327-342 (2017).

19 Romero, I. C. *et al.* Large-scale deposition of weathered oil in the Gulf of Mexico following a deep-water oil spill. *Environ Poll* **228**, 179-189 (2017).

20 Valentine, D. L. *et al.* Fallout plume of submerged oil from Deepwater Horizon. *Proc Natl Acad Sci USA* **111**, 15906-15911 (2014).

21 Bagby, S. C., Reddy, C. M., Aeppli, C., Fisher, G. B. & Valentine, D. L. Persistence and biodegradation of oil at the ocean floor following Deepwater Horizon. *Proc Natl Acad Sci USA* **114**, E9-E18 (2017).

22 Wang, Z., Fingas, M. & Page, D. S. Oil spill identification. *J Chromatography A* **843**, 369-411 (1999).

23 Reddy, C. M. *et al.* Composition and fate of gas and oil released to the water column during the Deepwater Horizon oil spill. *Proc Natl Acad Sci USA* **109**, 20229-20234 (2012).

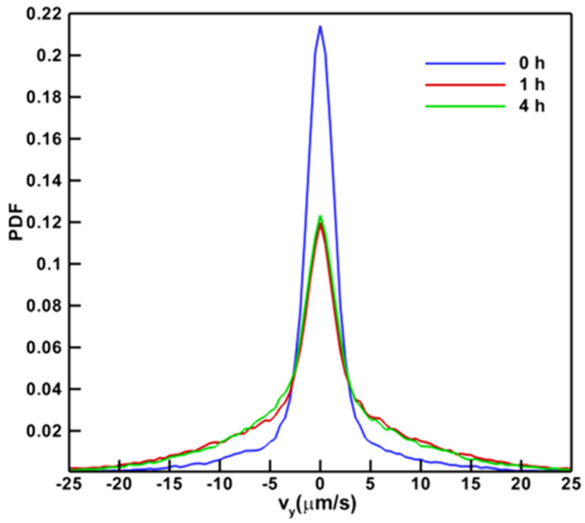

Fig. S1. The probability density (PDF) is plotted for bacteria velocity near an oil-water interface in otherwise quiescent fluid. Bacteria velocity PDF's at three times are shown according to the legend.

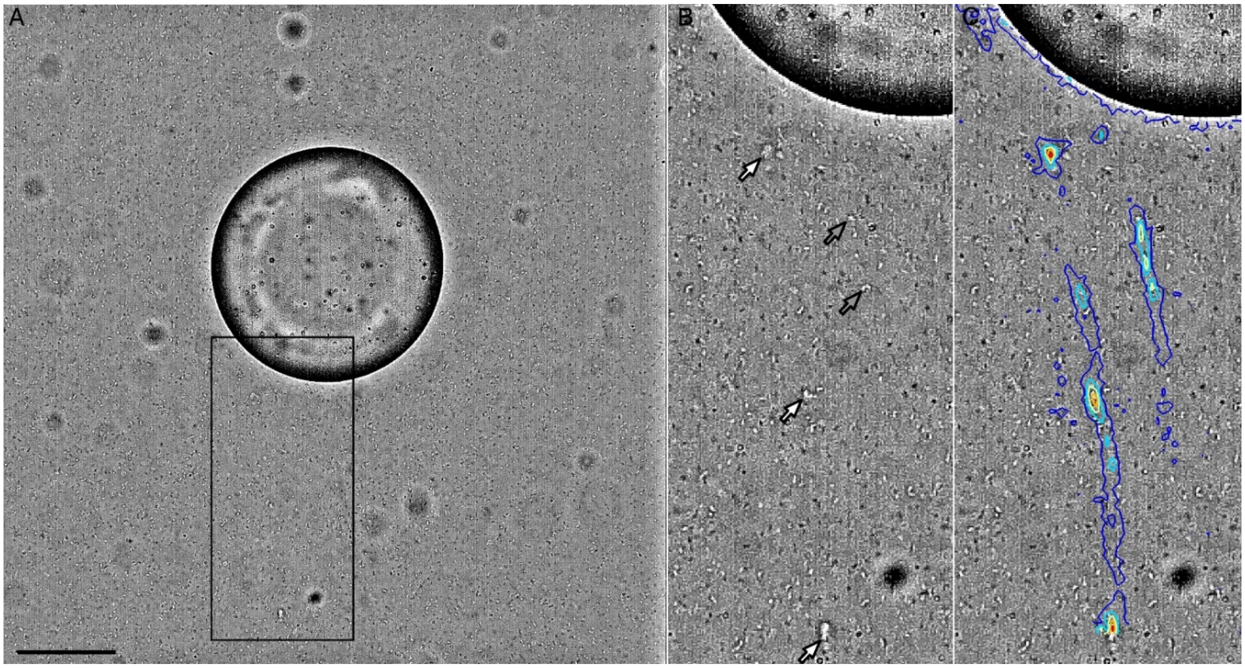

Fig. S2. (A) Sample micrograph of bacteria suspension around a droplet with two "invisible" streamer filaments. (B) Magnified micrograph showing the streamer filaments only identifiable with those cell clusters attached along them. Solid: filament 1. Hollow: filament 2. (C) The same micrograph as (B) superimposed by contours of normalized pressure gradient magnitude, $|\nabla^* p^*|$. The regions with elevated value coincides with locations of streamers and peaked at locations of attached cell clusters.

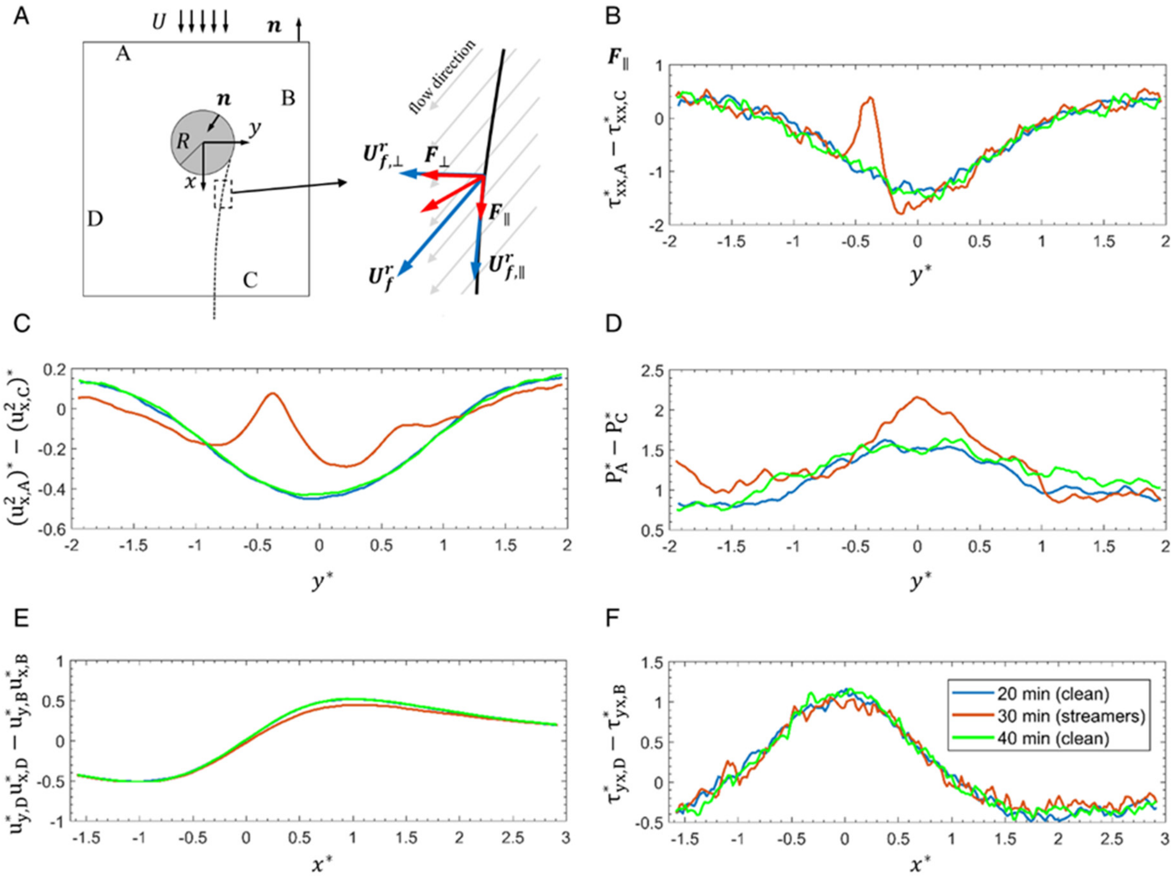

Fig. S3. Approximation of streamwise (x-) momentum budgets. (A) a 2D schematic of the flow past the pinned droplet is shown with surrounding control region ABCD and a streamer represented by the dotted line. In (A) the inset demonstrates the existence of both tangential ($\boldsymbol{F}_{\parallel}$) and normal ($\boldsymbol{F}_{\perp}$) forces on the streamer (black curve) due to flow ($\boldsymbol{U}_{\boldsymbol{f}}^{\boldsymbol{r}}$) crossing the streamer. In (B)-(F) the momentum budgets for sequences 20, 30 and 40 min after first exposure to bacteria are shown for experiment E7. The budgets shown are: B) the difference between the viscous normal stress crossing A and leaving C; C) the difference between x-momentum entering side A and leaving side C; D) the pressure difference between side A and side C; E) the difference in x-momentum crossing sides B and D; F) the difference in shear stress along sides B and D.

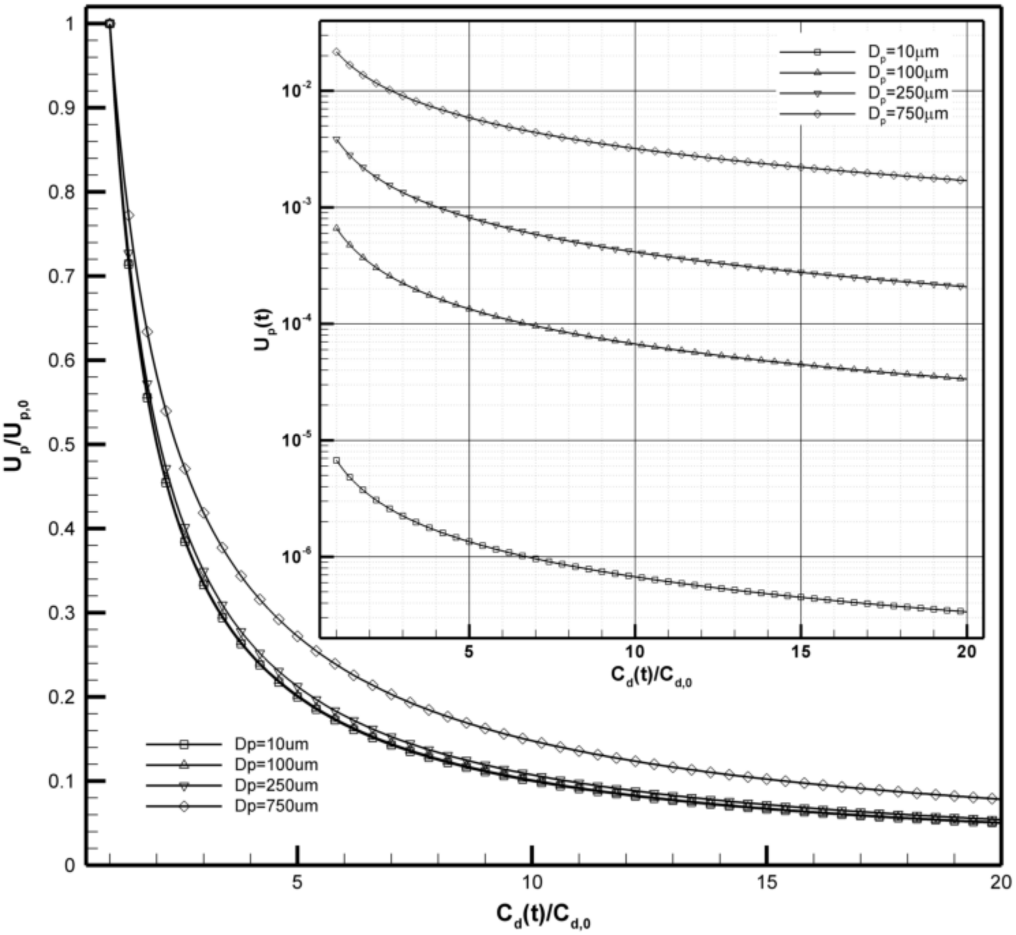

Fig. S4. Plots of the ratio of terminal velocities for a drop with streamers (U_p) to a drop without streamers ($U_{p,0}$) is plotted versus the ratio of drag coefficients for a drop with streamers (C_d) to a drop without streamers ($C_{d,0}$). The inset shows the absolute rising velocity of a drag versus its drag coefficient relative to a clean drop.

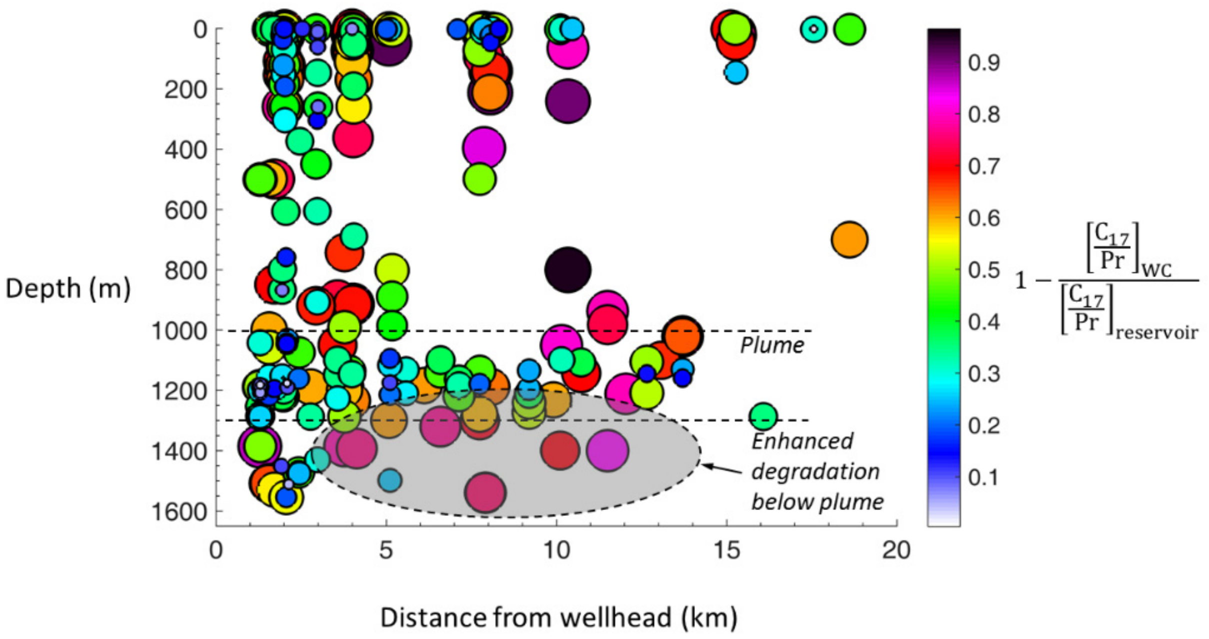

Fig. S5. A plot of the relative degradation of C_{17} versus depth (vertical axis) and distance from the wellhead (horizontal axis) is shown. Marker colors indicate the degree of degradation according to the color bar. Marker sizes additional increase with increasing degree of degradation.

Culture medium salinity	Washing medium	Velocity (µm/s)	D_b (10^{-9} m^2 /s)
	without wash	6.12±13.48	0.62
	Nutrient broth once	10.27±17.43	2.31
**25**	Nutrient broth twice	7.04±12.38	1.75
	25 ppt sea water once	7.03±13.06	1.4
	25 ppt sea water twice	8.36±16.47	1.43
	without wash	4.44 ± 8.82	0.71
	Nutrient broth once	4.18 ±7.32	0.58
**10**	Nutrient broth twice	11.31 ± 22.21	2.29
	10 ppt sea water once	8.43 ±20.20	1.64
	10 ppt sea water once	14.40±28.48	1.71
	Without wash	22.00 ± 28.87	2.26
**0**	Nutrient broth once	7.42 ± 10.22	1.58
	Nutrient broth twice	12.81 ± 18.13	5.05

Table S1. Mean bacterial swimming velocities and dispersion coefficients (D_b) for *Pseudomonas* P62 are tabulated for different washing protocols and culturing media (Difco, BD Cat. No. 234000).
